# Occupational Protection in Interventional Radiology. A Joint Guideline of the Cardiovascular and Interventional Radiological Society of Europe and the Society of Interventional Radiology

**DOI:** 10.1007/s00270-025-04195-4

**Published:** 2026-02-12

**Authors:** Donald L. Miller, Eliseo Vano, Stephen Balter, Cari M. Kitahara, Kevin A. Wunderle, Peter Reimer, Ryan F. Fisher, A. Kyle Jones, Beth A. Schueler, Graciano Paulo, Efstathios Efstathopoulos, Gabriel Bartal

**Affiliations:** 1 U.S. Food and Drug Administration, Silver Spring, MD USA; 2 Complutense University, Madrid, Spain; 3 Columbia University, New York, NY USA; 4 National Cancer Institute, Bethesda, MD USA; 5 Ohio State University, Columbus, OH USA; 6 Städtisches Klinikum Karlsruhe, Karlsruhe, Germany; 7 The MetroHealth System, Cleveland, OH USA; 8 The University of Texas MD Anderson Cancer Center, Houston, TX USA; 9 Mayo Clinic, Rochester, MN USA; 10 Escola Superior de Tecnologia da Saúde de Coimbra, Coimbra, Portugal; 11 National and Kapodistrian University of Athens, Athens, Greece; 12 AIMS.md, Kfar Saba, Israel

**Keywords:** Fluoroscopy, Computed tomography, Occupational protection, Ergonomics, Safety, Interventional radiology

## Abstract

**Purpose:**

Since the initial edition of this Guideline was published in 2010, it has become increasingly clear that occupational protection involves more than just radiation protection. Additionally, radiation exposure to operators and staff occurs from sources other than fluoroscopy. This Guideline has been updated to incorporate these topics and to reflect new information on occupational protection in interventional radiology.

**Methods:**

The CIRSE Standards of Practice Committee, in conjunction with SIR, established a writing panel of twelve clinicians, medical physics experts, and others with internationally recognised expertise in occupational protection in interventional radiology. The writing panel identified regulations, guidelines, and radiation protection documents from international and national sources, including governments, radiation protection organizations, and professional associations. Additionally, the writing panel performed a pragmatic search on PubMed for relevant studies published in English between 2011 and 2025. The final recommendations were developed by consensus.

**Results:**

The revised Guideline provides advice and guidance to physicians, radiographers, nurses, medical physics experts, and all other staff involved in interventional radiology. It also includes an online-only Annex with additional, in-depth coverage of several of the included topics.

**Supplementary Information:**

The online version contains supplementary material available at 10.1007/s00270-025-04195-4.

## Introduction

Interventional radiology is the medical specialty that uses image-guided techniques to diagnose, treat, follow up, and palliate a broad range of pathologies [1]. Occupational protection is a necessity in interventional radiology. These procedures may involve high radiation doses and dose rates for patients and staff, as well as a risk of musculoskeletal injury to staff [2–6]. Occupational radiation exposure in interventional procedures is closely related to patient exposure, so occupational radiation protection should be managed in an integrated way with patient protection. However, measures to protect staff must not impair the clinical outcome nor increase patient exposure [4, 7]. Occupational radiation protection requires the involvement of medical physics experts. Radiation protection measures must comply with local and national regulations and should consider the musculoskeletal detriment that can be caused by personal protective devices [4].

Radiation protection is necessary for all individuals working in the interventional radiology suite. This includes physicians, radiographers, and nurses [8–13], and individuals such as anesthesiologists who may be in a radiation environment only occasionally [14]. All these individuals may be considered radiation workers, depending on their level of radiation exposure and on national regulations. They require appropriate radiation dose monitoring, radiation protection tools and equipment, and education and training appropriate to their jobs [4, 15]. Their level of training should be based on the kind of work they do [15, 16].

Fluoroscopy is a major source of occupational radiation exposure in interventional radiology. It was addressed in the 2010 joint Cardiovascular and Interventional Society of Europe (CIRSE)—Society of Interventional Radiology (SIR) Guideline on Occupational Radiation Protection [7]. The current Guideline incorporates new information on occupational radiation protection and advice on other radiation sources, including CT guidance for procedures and radioembolization. It also provides a basic review of some relevant medical physics topics.

Since 2010 it has become increasingly clear that occupational protection involves more than just radiation protection. Interventional radiologists, like some other medical professionals, are at risk of developing chronic musculoskeletal injuries [6, 17–19]. These are discussed here. As with all operative procedures, there is also a risk of infection. This is not discussed here, as protection against infectious hazards is covered in detail elsewhere [20–23].

The goal of this Guideline is to provide advice and guidance to physicians, radiographers, nurses, medical physics experts, and all other staff involved in interventional radiology procedures. An online-only Annex provides additional information on many of the topics discussed here.

This Guideline and Annex use European terminology: radiographer (radiological technologist in the USA), and medical physics expert (medical physicist in the USA). Note also that in Europe a Radiation Protection Expert can be a nonmedical physics expert, as can a Radiation Safety Officer in the USA.

## Methodology

The members of the Writing Panel are experts in interventional radiology and medical physics across a broad spectrum of interventional procedures from both the private and academic sectors of medicine. They identified regulations, guidelines, and radiation protection documents from international and national sources, including governments [24–29], radiation protection organizations [1, 4, 15, 16, 30–54], and professional associations [6, 7, 20, 21, 23, 55–65]. In addition, and as appropriate, a critical review of peer-reviewed articles was performed with regard to their study methodology, results, and conclusions. The qualitative weight of these guidelines and articles was evaluated and used to write the document, such that it contains evidence-based data. Agreement was reached on all statements in this document and the accompanying Annex without the need for using consensus techniques.

The draft Guideline and Annex were critically reviewed by the CIRSE Executive Committee and separately by the SIR Executive Committee. The Writing Panel discussed the comments from both Executive Committees, and appropriate revisions were made to create the finished draft. This draft underwent Cardiovascular and Interventional Radiology’s standard peer review process. Appropriate revisions were made to create the finished document.

## Musculoskeletal Injuries

Awareness and concern regarding musculoskeletal injuries has increased among interventional radiologists since 2010 [5, 19, 66–68]. These injuries are not unique to interventional radiologists—they also occur in other interventionalists and in surgeons [6, 17, 18, 57, 61, 68–71]. They can occur in any individual who works in an interventional laboratory and are even more common in radiographers and nurses than in physicians [72]. They can result in pain, lost time from work, and burnout [5, 71]. Specific factors associated with back pain in interventionalists include repetitive motion patterns, insufficient recovery time, prolonged standing, axial loading of the spine, and awkward body postures. A static, uninterrupted posture will increase the risk of long-term back pain through sustained isometric contraction of the lumbar extensor muscle group [18]. Cervical pain is associated with the use of poorly positioned ceiling-mounted monitors and the repetitive head and neck movements required to view these monitors while performing procedures [6].

SIR and others have published recommendations to help prevent these injuries [4–6, 17, 18, 57, 61, 66–70, 73–75]. Enhancing ergonomic practices in interventional radiology is essential [19]. Suggested measures to help prevent musculoskeletal injuries are shown in Table 1 and discussed in detail in the Annex.

**Table 1:** Suggested measures to help prevent musculoskeletal injury in interventional radiology

Identify and stop performing activities that cause pain
Place the monitor in front of you at slightly below eye level [5, 6, 61]
Keep the monitor at a viewing distance that does not require you to bend forward to see image details
Adjust table height so that you do not need to bend over and your elbows are in a neutral position
Keep floor space clear to allow you to change positions during a case and to eliminate tripping hazards
Place the fluoroscopy pedal so that you do not need to move your body to reach it
Body positions that shift weight onto one leg should be avoided, even when using the foot pedal [18, 61]
Wear a lead apron that fits properly and provides only as much radiation protection as is needed
Use movable shields whenever possible to reduce the need for personal protective apparel
Avoid prolonged standing in one position
Avoid awkward body positions whenever possible
Take breaks of 1–2 min (“microbreaks”) every 20–60 min during a case [61, 69, 70]
Take breaks between cases whenever possible, to rest and stretch
Engage in physical fitness and conditioning programs to strengthen muscles and improve flexibility
Consider ergonomic issues when designing new fluoroscopy suites

## Radiation Effects

The potential effects of radiation exposure on radiologists have been reviewed relatively recently [76–79]. Radiation exposure may result in two types of adverse biological outcomes: stochastic effects and tissue reactions. Stochastic effects, including cancer and hereditary effects, are caused by a mutation or other permanent change in a cell that remains reproductively viable. The likelihood of a stochastic effect increases with dose (probably with no threshold, an assumption based on molecular knowledge of carcinogenesis) [51, 80]. Tissue reactions, formerly called deterministic effects, are caused by radiation-induced cell death or sterilization. They occur above a threshold radiation dose. Once the threshold is exceeded, there is a dose-related increase in severity.

Although the high levels of occupational exposure permitted before 1958 no longer occur [78], radiation-induced cancer remains the primary concern of most interventional radiology staff, as certain parts of organs and tissues (brain, lateral chest wall, and axillary portions of the breast) are not fully shielded and annual occupational exposures are generally higher for staff (particularly physicians) performing or assisting with interventional radiology procedures compared to most other medical radiation worker groups [13, 81, 82]. Radiation-induced cancer has been studied extensively [83]. Large, well-controlled observational cohort studies of occupationally exposed workers, including medical workers, with dosimetry data derived primarily from objective badge readings, have found modest associations between higher cumulative lifetime exposure to low-dose radiation and risks of death from solid cancers [84], including cancers of the breast [85] and lung [84, 86]. Stronger radiation dose-dependent associations have been observed with death due to leukemia [87, 88], but other high-quality studies found no association with leukemia incidence or death [89, 90]. Cohort studies have found no clear link between occupational radiation exposure and risk of malignant brain tumors, thyroid cancers, or other cancer outcomes [91–93]. For context, note that in the US population, approximately 40% of all individuals will develop cancer at some point in their life, and 20% of individuals will have cancer as their cause of death [94].

Occupational radiation exposure in the interventional radiology suite may also be linked to cytogenetic changes (e.g., chromosomal aberrations) [79] and certain tissue reactions [35], including cataracts [95–97], cardiovascular effects [98], and neurologic effects [99]. These have been studied extensively as well [35, 49, 83]. High doses to the hands can occur when poor technique is used during administration of radioisotopes during radioembolization, during CT-guided interventions, or during fluoroscopy if the hands are placed in the direct beam. With CT fluoroscopy in particular, the annual dose limit for the hands can be reached after fewer than 10 procedures if the hands are kept in the beam [4, 100]. Individuals who routinely place their hands in the direct beam may violate local regulations [53] and are at risk of developing chronic tissue reactions. A more extensive discussion of stochastic effects and tissue reactions is provided in the Annex.

## Occupational Dosimetry

### Dosimeter Use

Dosimeters worn by interventional radiology workers are typically collected and analyzed monthly to provide timely detection of elevated exposure levels and allow for prompt intervention and mitigation. Occupational dose information cannot be accurate unless dosimeters are worn routinely and correctly. Both single- and double-dosimeter protocols are in common use. Single-dosimeter measurements use one dosimeter, worn outside of protective apparel at the collar, to estimate whole-body and eye exposure. The two-dosimeter method adds a second dosimeter, worn inside protective apparel at the waist. The dose to the lens of the eye may be estimated from the collar dosimeter. The International Commission on Radiological Protection (ICRP) and National Council on Radiation Protection and Measurements (NCRP) recommend that interventionalists use the two-dosimeter method because the weighted average of the two readings provides a better estimate of whole-body exposure [4, 47]. More than half of the European countries that responded to a European Radiation Dosimetry Group survey have legal requirements about the number and position of dosimeters used for estimation of the effective dose when radiation protection garments are used [101]. In some countries, the required locations for the dosimeters differ from those in the standard two-dosimeter method. Local and national regulations for occupational dose monitoring must be followed, but with either method an additional badge may be worn if indicated. Additional dosimeters for the hands may be especially appropriate for specific procedures, such as radioembolization and CT fluoroscopy, but are of value for all procedures [4]. If doses to the collar dosimeter are expected to be high, an additional dosimeter near the operator’s eyes may be recommended. This is particularly useful when radioprotective eyewear is used, as estimates based on the collar dosimeter may overestimate dose to the lens of the eye. Additional information on dosimeters and dosimeter use is provided in the Annex.

Adherence to institutional and regulatory standards is essential. ICRP Publications 60, 103, and 139 and NCRP Reports Nos. 122, 168, and 187 provide background information and guidelines specifically for managing occupational exposure methods [4, 30, 34, 43, 47, 52]. These reports recommend the use of dosimeters, regular monitoring, and strict adherence to dose limits. In interventional radiology facilities, optimal practice for occupational radiation protection includes the involvement of a Radiation Protection Expert or medical physics expert with knowledge of the practical aspects of interventional radiology and the fluoroscopes in clinical use.

### Occupational Dose Limits

Effective dose (*E*) is intended to be proportional to the risk of radiation-induced detriment in health, principally from cancer. Occupational dose limits in Europe are included in Council Directive 2013/59/Euratom [24] and are based on ICRP recommendations [34, 35]. In the USA, effective dose equivalent (EDE) is used for these dose limits, which are also called Maximum Permissible Doses (MPD). The differences are described in the Annex. Occupational dose limits for Europe and the USA are summarized in Table 2. Additional restrictions apply to the occupational exposure of pregnant workers. These are discussed below, in the section on Pregnancy.

**Table 2:** Current dose limits (maximum permissible doses) for occupational exposure in Europe per 2013/59/Euratom [24] and the USA

Dose quantity	Region and dose limit
	**Europe**
Effective dose	
Annual	20 mSv/y averaged over five consecutive years (100 mSv in 5 years), with no single year exceeding 50 mSv
Equivalent dose	
Lens of the eye	20 mSv/y, averaged over defined periods of five years, with no single year exceeding 50 mSv
Skin^a^	500 mSv/y
Extremities (hands and feet)	500 mSv/y
Fetus	1 mSv for the entire pregnancy
	**USA**
EDE	
Annual	50 mSv/y
Cumulative	10 mSv x age (y)
Equivalent dose	
Lens of the eye	150 mSv/y
Skin^a^	500 mSv/y
Extremities (hands and feet)	500 mSv/y
Fetus	5 mSv over the duration of the pregnancy

*EDE* effective dose equivalent

^a^Averaged over 1 cm^2^ of the most highly irradiated area of the skin

Occupational dose limits for the lens of the eye have historically been based on the premise that cataract formation is observed only at high doses. The US Nuclear Regulatory Commission limit for lens exposure is 150 mSv/y and has not changed since 1991. In 2011, the ICRP recommended an annual equivalent dose limit for the lens of the eye of 20 mSv, with no single year exceeding 50 mSv, based on the assumption of a dose threshold of 500 mGy [35]. In 2016, the NCRP lowered its recommended dose limit for the lens of the eye from an effective dose of 150 mSv to an absorbed dose of 50 mGy, with the justification that the 50 mGy limit (as opposed to the 20 mSv limit recommended by the ICRP) is adequately protective and less restrictive for radiology practice [102]. However, in light of findings from epidemiologic studies over the last decade [95], others have suggested that even the lower ICRP recommended limits may be too lenient [103].

### Occupational Doses in Interventional Radiology

A busy interventional radiologist who takes all appropriate radiation safety precautions is unlikely to exceed the 20 mSv ICRP limit for annual exposure. Several studies indicate that such an individual is more likely to have an *E* of 2–6 mSv/y [4, 9, 82, 104]. In France, all medical workers have shown a trend toward decreasing annual occupational dose [11]. In the UK, an overall downwards trend was also observed, with few physicians exceeding 6 mSv/y [14]. Although collar badge dose decreased in radiologists, there was an apparent increase in lens of the eye dose. The reason is not clear. This was not seen in a study of US medical staff performing or assisting with fluoroscopically guided interventional (FGI) procedures [81, 82]. Radiographers in the USA have a median annual dose < 2 mSv, and are unlikely to exceed 10 mSv, with a trend to decreasing annual doses in recent years [13]. Nurses tend to have lower annual doses than radiographers, < 1 mSv [9].

## Personal Protective Equipment

Staff exposure in interventional radiology is mainly due to scattered radiation originating from the patient. Of the radiation emitted by the X-ray tube during fluoroscopic procedures, approximately 80% is absorbed by the patient’s body, about 20% is scattered in different and random directions, and only 1% reaches the detector [105, 106]. The scatter field is highly asymmetric and is approximately 10 times more intense on the tube side of the patient than on the image receptor side [107]. Scatter distribution maps are provided in the documentation supplied with International Electrotechnical Commission (IEC) compliant fluoroscopes (essentially all fluoroscopes sold in the USA and Europe) [108]. Occupational radiation protection in fluoroscopy is achieved using a combination of personal protective equipment (PPE) that includes garments such as aprons, vests, skirts, thyroid shields, and eyewear; additional devices such as fluoroscope-mounted, rolling, or ceiling-suspended shields; and structural shielding incorporated into the walls, windows, and doors of the procedure room [109]. Additional information on PPE and radioprotective shields is provided in the Annex.

PPE incorporates highly attenuating materials. These include lead or other high density, high atomic number (Z) materials [110]. For lead-free garments, the K-edge absorption characteristics of moderate Z materials are used, often in bilayers [111, 112]. Some countries no longer allow lead in PPE because lead must be managed as a hazardous material. Protective value is commonly stated in terms of “lead equivalence,” but transmission at specific radiation qualities is preferred because “lead equivalence” varies depending on scatter radiation quality [113]. At tube energies above 90 kV, lead-free materials are less protective than lead [110]. They may be adequate for protection during fluoroscopy but are less effective for protection during CT-guided procedures due to the higher kV used in CT. Typical 0.5 and 0.35 mm nominal thickness garments transmit 5% and 10% of fluoroscopic scatter. Unless national or local regulations specify a minimum lead or lead equivalent thickness, an appropriate thickness should be based on the wearer’s under-apron dosimeter readings [4, 47].

Staff work habits and positioning should be accounted for when purchasing PPE. Some wraparound garments only provide the nominal protection where the two layers overlap. Ancillary staff who spend substantial time with their back to the patient should be aware of this. Garment weight is proportional to its protective value. Additional aspects of apron design and use are discussed under Musculoskeletal Injuries and Pregnancy in this Guideline and in the Annex.

Radioprotective eyewear is necessary for those scrubbed in for interventional radiology procedures where ionizing radiation is used. These are available in a range of nominal lead thicknesses and anatomic coverage [4]. Radioprotective eyewear is also available for workers who need vision correction. To maximize protection, eyewear should have a large surface area, should extend laterally along the side of the face, and should fit tightly to the face [97, 114–116]. Eyewear needs to be comfortable, as it should be worn routinely. With appropriate design, eyewear with lower lead content can be both lighter and more effective than eyewear with a higher lead content [97, 116]. Work habits and positioning should also be accounted for, as turning one’s head away from the patient toward display monitors can drastically increase radiation scattered to the eyes [114]. Radiation to other tissues in the head results in secondary scatter [117]. Protective eyewear cannot eliminate radiation dose to the lens of the eye from this secondary scatter. Use of ceiling-suspended shields is still necessary [118].

The cranium attenuates scattered radiation and reduces radiation dose to the brain by 40 to 50% [119, 120]. Radioprotective caps have been proposed to further reduce radiation exposure of the brain and the lens of the eye [119]. Caps that only cover the head above the level of the eyes do not protect against scattered radiation from the patient [64, 109, 121–124]. Some “caps” have ear flaps or extend to cover the head and neck, except for the face. These can provide some additional protection to the brain but may cause discomfort or interfere with hearing [121, 125].

Radioprotective surgical gloves and radioprotective creams are intended to protect the wearer’s hands from scatter while the hands are outside the useful beam. For systems with automatic dose rate control, placing one’s hand in the beam while wearing these gloves or creams increases both wearer and patient dose, as the system is attempting to penetrate two layers of leaded protection, while only a single layer is protecting the wearer [126]. 

Because approximately 70% of radiation-induced DNA damage is due to reactive oxygen species and free radicals created during X-ray irradiation, there is research interest in the use of oral antioxidants such as ascorbic acid, N-acetylcysteine, and beta carotene to prevent this damage [127–130]. Some protective effect on the DNA in circulating mononuclear cells has been observed in patients given oral antioxidants prior to nuclear medicine bone scans or cardiac catheterization, but results are mixed [127, 130]. Prophylactic dietary antioxidant supplementation does not appear to have been studied in health care workers regularly exposed to low doses of ionizing radiation.

## Pregnancy

Pregnant radiation workers often worry about potential radiation risks to their unborn child. Inaccurate knowledge or misinformation can create a barrier for women who are considering careers in this field, possibly resulting in missed career opportunities. Radiation exposure during pregnancy does pose risks, including pregnancy loss, congenital malformations, developmental delay, and carcinogenesis, but these risks are extremely small with the use of appropriate radiation protection equipment and methods. These risks can be tissue effects (intrauterine growth retardation, miscarriage, and congenital defects) or stochastic effects (childhood cancer). The same risks occur in pregnancies where the embryo/fetus was never exposed to radiation (Table 3).

**Table 3:** Spontaneous pregnancy risks in the general population. Reprinted with permission from [58]

Type of risk	Spontaneous risk^a^
Risk of very early pregnancy loss (before first missed period)	~ 1 in 3
Risk of spontaneous abortion in known- pregnant women	~ 1 in 7
Risk of major congenital malformations	~ 1 in 33
Risk of severe mental disability	~ 1 in 200
Risk of childhood leukemia per year	~ 1 in 25,000
Risk of early-or late-onset genetic diseases	~ 1 in 10
Prematurity	~ 1 in 25
Growth retardation	~ 1 in 33
Stillborn	~ 1 in 50–250
Infertility	~ 1 in 15 couples

^a^Spontaneous risks facing an embryo at conception (i.e., at a 0 mGy radiation dose)

Reprinted from Journal of Vascular and Interventional Radiology, Vol. 26(2), Dauer LT, Miller DL, Schueler B, Silberzweig J, Balter S, Bartal G, et al., Occupational radiation protection of pregnant or potentially pregnant workers in IR: a joint guideline of the Society of Interventional Radiology and the Cardiovascular and Interventional Radiological Society of Europe. Journal of Vascular and Interventional Radiology, Pages 171–81, Copyright 2015, with permission from Elsevier

Biological effects on the fetus depend on the stage of pregnancy and the absorbed dose. The risks are greatest during organogenesis and the initial embryonic period in the first trimester of pregnancy, lower during the second trimester, and even lower in the third trimester [48]. The most critical period for the occurrence of tissue effects is between 8 and 15 weeks of gestation, when the radiation dose threshold is approximately 100 mGy [48, 58]. This is much higher than both regulatory limits and the expected level encountered by radiation workers using protective measures. Stochastic effects are thought to have no threshold dose, but the embryo/fetus is considered to have a similar risk for potential carcinogenic effects of radiation as an infant. Overall, there may be an extremely small increase in the probability of congenital abnormalities or childhood cancer with occupational exposure within the expected dose to the fetus or embryo (Table 4) [31, 58, 131].

**Table 4:** Probability of a live birth without malformation or without childhood cancer as a function of radiation dose. Reprinted with permission from [58]

Dose to embryo/fetus	No malformations (%)	No childhood cancer (%)	Neither (%)
0 mSv	96.00	99.93	95.93
0.5 mSv	95.999	99.926	95.928
1.0 mSv	95.998	99.921	95.922
2.5 mSv	95.995	99.908	95.91
5.0 mSv	95.99	99.89	95.88
10.0 mSv	95.98	99.84	95.83
50. mSv	95.90	99.51	95.43
100.0 mSv^a^	95.80	99.07	94.91

^a^For conceptus doses > 100 mSv, consult a qualified medical physicist/medical physics expert for risk estimates

Reprinted from Journal of Vascular and Interventional Radiology, Vol. 26(2), Dauer LT, Miller DL, Schueler B, Silberzweig J, Balter S, Bartal G, et al., Occupational radiation protection of pregnant or potentially pregnant workers in IR: a joint guideline of the Society of Interventional Radiology and the Cardiovascular and Interventional Radiological Society of Europe. Journal of Vascular and Interventional Radiology, Pages 171–81, Copyright 2015, with permission from Elsevier

Radiation dose to the embryo/fetus is monitored for radiation workers using a dosimeter worn at waist level under any protective apparel. The dosimeter should be replaced monthly, starting when the worker declares her pregnancy. When two-dosimeter monitoring systems are used, the monitor worn under radiation protective garments should be placed at the waist level [48]. It is advisable to monitor radiation levels in a similar fashion when planning a pregnancy, to determine the likely embryo/fetus radiation dose for an individual’s expected workload. Real-time dose monitoring during pregnancy can also be beneficial by offering regular updates on radiation dose levels.

Recent studies indicate that the occupational dose to the embryo/fetus of interventional radiology staff is well below 1 mSv. To date, there is no evidence in humans of congenital abnormalities at this level of dose [65]. Wunderle et al. evaluated fetal dose for the declared pregnancy for all declared pregnant workers in all imaging departments (including interventional radiology and interventional cardiology) over a 9-year period [132]. The average fetal dose was < 0.3 mSv. Meek et al. found that the waist-level measurements for two pregnancies of one interventional radiology physician averaged 0.47 mSv for the entire 40 weeks of each pregnancy and 0.135 mSv for 10 pregnancies in 10 nonphysicians (maximum 0.38 mSv) [133]. Fetterly et al. reported a median under-apron abdomen measurement of 0.22 mSv (95th percentile 0.8 mSv) for a 40-week period collected from 42 interventional cardiology and electrophysiology physicians who wore 0.5 mm lead equivalent aprons [134]. Similarly, in a study involving an interventional radiology fellow who performed 280 neurointerventional procedures while wearing two 0.5 mm lead apron skirts, the radiation badge worn under the skirts recorded 0 mSv during the six months of declared pregnancy [135].

Pregnant workers who desire additional radiation protection for their conceptus sometimes wear an additional lead apron or a maternity apron. This could decrease conceptus dose compared with a standard lead apron, but its additional weight may cause significant fatigue and strain during lengthy procedures [58]. It can also increase the potential for, or exacerbate, musculoskeletal and back pain, which is commonly encountered during a normal pregnancy even when no apron is worn.

In Europe, occupational radiation exposure during pregnancy is regulated by directives from the European Commission, which are reflected in national laws. European Directive 2013/59/Euratom mandates that after a worker has declared her pregnancy, the fetal dose should not exceed 1 mSv [24]. This aligns with ICRP recommendations [34]. The International Atomic Energy Agency (IAEA) states that “Notification of the employer by a female worker if she suspects that she is pregnant or if she is breast-feeding shall not be considered a reason to exclude the female worker from work” [37], but in some European countries (e.g., Austria, Hungary, Italy), occupational radiation exposure during pregnancy is not allowed [63]. In the USA, NCRP recommends a monthly dose limit of 0.5 mSv equivalent dose for the embryo/fetus after pregnancy is declared [50]. Moreover, pregnant radiation workers in the USA are not required to declare their pregnancy to their employer due to legislation and legal rulings meant to protect pregnant women from employment discrimination [29, 60]. This is in line with IAEA guidance that “a female worker cannot be compelled to notify her employer if she is aware or suspects that she is pregnant” [38].

In summary, legal requirements vary among countries, but pregnant interventional radiology physicians and staff can continue working safely during pregnancy if they follow appropriate radiation protection practices [64]. Medical physics experts should estimate the radiation dose to the conceptus for pregnant workers. Use of appropriate radiation protection equipment and methods should always result in exposure to the fetus well below the regulatory level. Termination of pregnancy because of fetal radiation exposure of < 100 mGy is not justified [48, 65], but this is rarely an issue because fetal doses should be less than 1 mGy, even with moderate to heavy workloads. Wearing protective garments with greater than 0.5 mm lead equivalent thickness provides no meaningful advantage for fetal radiation protection and increases the risk of musculoskeletal injury [64].

## Practical Advice to Reduce or Minimize Occupational Radiation Dose

In general, decreasing patient dose results in a proportional decrease in scatter dose to the operator, making patient dose reduction beneficial for both patient and practitioner, creating a “win–win” scenario [4, 7]. Practical advice to reduce or minimize occupational radiation dose in specific interventional radiology settings is provided here and discussed more extensively in the Annex.

### Fluoroscopically Guided Procedures

Modern interventional fluoroscopy systems are extremely complex and highly sophisticated. Operators have both direct and indirect control over various factors that influence radiation dose as well as image quality. One of the most critical decisions an operator makes at the start of any procedure is selecting the appropriate imaging protocol on the fluoroscope. Optimizing these imaging protocols for specific clinical tasks or groups of tasks should be a collaborative effort among the clinical team, including an appropriately trained medical physics expert and a vendor imaging specialist.

The imaging protocol refers to the software exam type used during the procedure—denoted by some vendors as “organ programs” or “exam sets.” Modern systems offer imaging protocols with 100 or more individual parameters, each with multiple settings, resulting in a vast array of possible combinations. These settings influence key aspects such as the X-ray beam spectrum (which affects low contrast resolution and radiation dose rates), focal spot size selection (which affects high contrast resolution), and technical parameters that directly influence radiation dose rates, including default pulse rate, pulse width, spectral filtration, detector dose level, and maximum allowable dose rate (e.g., low, normal, or high fluoroscopy dose limits). The imaging protocol also determines the image processing applied to the acquired images, which must be appropriately tuned to the other parameters.

The operator is responsible for protecting others in the procedure room. In a randomized controlled trial, Komemushi et al. [8] showed that when nurses told the operator that they were going to approach the patient, the operator could avoid fluoroscopy while the nurse was close to the patient. This resulted in nurses having significantly lower occupational radiation doses, while operator doses were not significantly different. Additional parameters and techniques directly controlled by the operator are listed in Table 5 and described in more detail in the Annex. This information has not changed over time [7, 32, 47, 136].

**Table 5:** Techniques to reduce occupational dose during fluoroscopically guided procedures

Obtain appropriate training
Use appropriate fluoroscopic imaging equipment
Use protective shielding
Wear your dosimeters and know your own dose
Use all available information to plan the interventional procedure
Use guidance tools (e.g., navigation systems, fluoroscopy overlays, vessel tracking) to help decrease procedure time and radiation for the procedure
Use available patient dose reduction technologies
Minimize fluoroscopy time
Minimize the number of radiographic images (e.g., digital subtraction angiography)
Use good imaging-chain geometry
Collimate
Position yourself in a low scatter area
Step out of the room during long radiographic acquisitions, especially cone-beam CT

### Radioembolization

In radioembolization procedures, microspheres containing a radioactive isotope are injected into hepatic artery branches to deliver localized, high doses of radiation to hepatic tumors. Currently, microspheres use ^90^Y, a beta emitter. As of April 2025, ^166^Ho, a beta and gamma emitter, is not available. Most of the radiation exposure to staff in radioembolization procedures is from fluoroscopy. The radionuclide in use contributes only a small additional exposure during specific portions of the procedure. However, as discussed in the Annex, the operator can receive high finger doses if appropriate protection measures are not used.

The most critical step in terms of occupational radiation exposure is microsphere injection. All vials containing ^90^Y or ^166^Ho activity, and all instruments and disposable items used for preparing the dose and implanting the device, should be handled with forceps and appropriate shielding to reduce finger doses. Due to the high-energy beta emission, shielding is best provided with a low atomic number material such as polymethylmethacrylate [4]. Double gloves are recommended to allow removal of a contaminated outer glove with a gloved hand. It is essential to flush all tubes and catheters with water or saline for injection before manual manipulation. Drescher et al. [137] showed that for ^166^Ho administration, microsphere accumulation occurred at the three-way stopcock and the microcatheter hub. Disconnecting and reusing microcatheters for injection into a second or third location is a potential risk, so use of a new microcatheter may be desirable. In addition to all technical measures of radiological protection, training to speed up all steps of the procedure leads to a significant reduction of occupational exposure [4].

### CT-Guided Procedures

CT-guided interventions may be performed with intermittent CT, often referred to as “quick-check” or “step and shoot,” or CT fluoroscopy. For occupational protection, intermittent CT is preferable, as staff may step into the control area and benefit from structural shielding or stand in the shadow of the CT gantry [59]. With CT fluoroscopy, the need to manipulate instruments during CT scanning exposes the operator to relatively high levels of backscattered radiation. Fortunately, real-time CT fluoroscopy is not necessary for most CT-guided procedures [138]. Intermittent CT fluoroscopy is usually adequate. Use of this technique results in substantially decreased CT fluoroscopy times and radiation dose as compared to CT fluoroscopy and avoids direct exposure of the operator’s hand [138, 139].

Regardless of the method used, limiting the length of CT scans, using task-specific baseline technical factors, and adapting technical factors to patient size (e.g., using tube current modulation) will help optimize radiation doses to both the patient and the operator. The use of ultrasound as an adjunct or even as a replacement for CT is a further consideration. In many cases, prior information available from diagnostic imaging studies can be used to plan a CT-guided procedure, e.g., precisely define a limited pre-procedure planning scan range. As shown in Table 6, there are many methods for limiting occupational exposure during CT-guided interventions [59, 100, 138–143]. Further discussion of these methods is provided in the Annex.

**Table 6:** Methods for limiting occupational exposure during CT-guided interventions

Avoid CT fluoroscopy; use the “quick-check” (“step and shoot”) CT imaging mode instead
Consider ultrasound as an adjunct or replacement for CT
Use information from previous imaging studies to plan the procedure and limit the scan range
Use task-specific baseline technical factors
Limit the length of helical scans
Step into the control room whenever possible during CT acquisitions
Stand in the shadow of the gantry during short CT acquisitions
Use mobile shields to provide additional protection for individuals who need to remain in the CT room during scanning
Use angular beam modulation during CT fluoroscopy, if available
If real-time CT fluoroscopy is necessary, stand as far as possible from the scan plane, use a long needle holder and a table-mounted lower body shield
Single-use sterile protective drapes may be useful but need to be placed close to, but not overlapping, the primary beam
Sterile radioprotective gloves offer limited protection at the beam energies used in CT
PET^a^ radiation contributes only a small fraction to patient radiation doses and personnel exposure during PET/CT-guided procedures

^a^*PET* positron emission tomography

## Training

All individuals who participate in interventional radiology procedures need appropriate initial and periodic training [1, 15, 16, 25]. Here, “training” means the applied knowledge and practical aspects of radiation protection that result in improved efficiency and productivity. While education is related to theoretical knowledge, training is more practical and is usually conducted in a clinical environment [16, 144].

Safely delivering patient care in interventional radiology involves teamwork among individuals with a wide range of skill sets and professional backgrounds. Both patients and their health care teams are exposed to several risks relating to the delivery of these procedures. Appropriate training contributes to reducing patient and staff risks [145–147]. Effective radiation management is one part of a general safety culture [39], a concept that should be integrated into all radiation protection training [148, 149]. Real or potentially unsafe situations for patients and staff (not just radiation) can occur. Any individual who observes the situation should be able to communicate their concern appropriately to coworkers without fear of retaliation, intimidation, harassment, or discrimination. To ensure that the message is heard, the recipient should acknowledge and appropriately respond. All interventional radiology staff, including physicians, should be trained in this aspect of communication, which is an important part of a safety culture [39].

Member states of the European Union are required to promote and define radiation protection education and training for health professionals, but a recent survey showed uneven compliance [150]. This is not unique to Europe [40]. The ICRP, IAEA, European Commission, and NCRP recommend additional training elements for physicians who perform interventional procedures, beyond the level of training for other physicians [1, 15, 16, 25, 32]. Additional training is also needed for physicians who perform pediatric interventions due to the greater radiosensitivity of these patients compared to adults [15].

Some parts of radiation management training could incorporate virtual components, such as virtual laboratory exercises, simulators, and virtual reality (VR). Being able to “see” the radiation fields as the task progresses can provide a cognitive link between how a clinical task is performed and its radiation effects on both patients and staff. Students generally feel that they can recognize the clinical relevance of concepts taught via VR much more easily than those taught via lectures [151]. VR and simulation are discussed in more detail in the Annex.

Education and training may be provided by individual facilities, outside academic institutions, health and regulatory agencies, professional bodies, equipment suppliers, freestanding training groups, or others [16]. Training content should be periodically reviewed and refreshed to ensure that it remains current. Accreditation of training suppliers and training content should be considered. This is required in some European countries. In this context, “accreditation” means that a training supplier has been approved by an appropriate body to provide education or training [16]. Additional information on training is provided in the Annex.

## Management Responsibilities

Administrators are unlikely to have a background in interventional radiology. Formal and informal training in interventional radiology, including a review of the risks to patients and workers, should be provided to these individuals.

Optimizing staff safety goals without compromising patient care is vital. Medical facilities should have a formal group whose task is to evaluate all risks to the facility, its patients, and workers. This group should have sufficient access to worker radiation safety experts to facilitate its responsibilities for worker risks in interventional radiology, including cataracts, radiogenic cancer, and the musculoskeletal risks associated with the use of personal radioprotective devices. Current radiation protection devices and fluoroscopy suite design contribute to musculoskeletal injuries in interventional physicians, nurses, and radiographers [5, 17, 19, 57, 72]. It is possible to reduce risk to a reasonable level without using excessive resources [4, 152].

Worker radiation safety in interventional radiology is a responsibility of the designated health professional (“Radiation Protection Expert” or “Radiation Protection Officer” in Europe, or “Radiation Safety Officer” in the USA). At some facilities in the USA, the Radiation Safety Officer’s responsibility is limited to oversight of radioactive materials. Medical physics experts may also have responsibilities for worker safety. Support from these individuals varies widely from the minimum of assuring regulatory compliance to full integration. All these individuals are more effective in optimizing safety if they have an appropriate understanding of the medical conduct of interventional procedures.

## Glossary

**Absorbed dose**: the energy imparted to matter by ionizing radiation per unit mass of irradiated material at the point of interest. In the Système Internationale (SI), the unit is J kg^–1^ with the special name gray (Gy) [47].

**Accreditation:** with respect to radiation protection training, accreditation means that a training supplier has been approved by an appropriate body to provide education or training [16].

**Cohort study:** a research study that compares a particular outcome (such as lung cancer) in groups of individuals who are alike in many ways but differ by a certain characteristic, such as occupational radiation exposure. In a cohort study setting, random errors in estimates of dose tend to drive an association toward null (no effect) rather than to artificially inflate, or induce, an association.

**Detriment**: the total harm to health experienced by an exposed group and its descendants because of the group’s exposure to a radiation source. Detriment is a multidimensional concept. Its principal components are the stochastic quantities: probability of attributable fatal cancer, weighted probability of attributable nonfatal cancer, weighted probability of severe heritable effects, and length of life lost if the harm occurs [34].

**Dose equivalent**: a measure of the biological damage to living tissue as a result of radiation exposure. Also known as the “biological dose,”, the dose equivalent is calculated as the product of absorbed dose in tissue multiplied by a quality factor and then sometimes multiplied by other necessary modifying factors at the location of interest. The dose equivalent is expressed numerically in rems or sieverts (Sv) [27].

**Dose limit**: the value of the effective dose or the equivalent dose to individuals from planned exposure situations that shall not be exceeded [34]. The intent is to prevent the occurrence of radiation-induced tissue reactions or to limit the probability of radiation-related stochastic effects.

**Effective dose**: the tissue-weighted sum of the equivalent doses in all specified tissues and organs of the body, given by the expression:

$$E = \mathop \sum \limits_{T} w_{T} \mathop \sum \limits_{R} w_{R} D_{T,R} {\mathrm{or}}\,E = \mathop \sum \limits_{T} w_{T} H_{T}$$

where *H*_*T*_ or *w*_*R*_ *D*_*T,R*_ is the equivalent dose in a tissue or organ, T, and *w*_*T*_ is the tissue weighting factor. The unit for effective dose is the same as for absorbed dose, J kg^−1^, and its special name is sievert (Sv) [34]. Effective dose (E) applies only to stochastic effects.

**Effective dose equivalent**: the sum of the products of the dose equivalent to the organ or tissue (${H}_{T}$) and the weighting factors (${w}_{T}$) applicable to each of the body organs or tissues that are irradiated (*H*_*E*_ = Σ ${w}_{T}{H}_{T}$) [27].

**Equivalent dose (H**_***T***_**):** the mean absorbed dose (*D*_*T,R*_) in a tissue or organ *T* weighted by the radiation weighting factor (*w*_*R*_) for the type and energy of radiation incident on the body:

$$H_{T} = \mathop \sum \limits_{R} w_{R} D_{T,R}$$

The unit of organ equivalent dose is J kg^−1^ and has the special name sievert (Sv).

**Excess relative risk**: the rate of disease in an exposed population divided by the rate of disease in an unexposed population, minus 1.0. This is often expressed as the excess relative risk per Gy or per Sv [34]. (See also relative risk.)

**Fluoroscopically guided interventional procedure**: an interventional diagnostic or therapeutic procedure performed via percutaneous or other access routes, usually with local anesthesia or intravenous sedation, which uses external ionizing radiation in the form of fluoroscopy to: localize or characterize a lesion, diagnostic site, or treatment site; monitor the procedure; and control and document therapy [47].

**Gray**: the special name for the SI unit of absorbed dose: 1 Gy = 1 J kg^−1^ [34].

***K*-edge**: the binding energy of the innermost electron shell (*K*-shell) of an atom. There is a marked increase in X-ray absorption of X-rays whose energy is just above the *K*-edge due to the photoelectric effect.

**Interventional radiology**: the medical specialty that uses image-guided techniques to diagnose, treat, follow up, and palliate a broad range of pathologies [1].

**Maximum permissible dose**: in the USA, a regulatory dose limit.

**Occupational radiation exposure**: radiation exposures to individuals that are incurred in the workplace because of situations that can reasonably be regarded as being the responsibility of management (radiation exposures associated with medical diagnosis of or treatment for the individual are excluded) [47].

**Odds ratio**: the ratio of the odds of an event occurring in the exposed group to the odds of the event occurring in the nonexposed group. It is commonly used in case–control studies where the incidence rates of the outcome are not directly measured, but given the outcome the odds of exposure can be calculated.

**Operational quantities**: quantities used in practical applications for monitoring and investigating situations involving external exposure. They are defined for measurements and assessment of doses in the body [34].

**Personal protective equipment**: garments and devices worn to protect against radiation exposures, such as aprons, thyroid shields, and leaded eyewear.

**Posture**: the spatial arrangements of body parts as they align to perform a task.

**Rad**: a unit of absorbed dose. One rad is equal to an absorbed dose of 100 ergs/gram or 0.01 J/kg (0.01 Gy) [28]. For X-rays and gamma rays, 1 rad = 1 rem = 10 mSv.

**Relative risk**: the ratio of the probability of an event occurring in the exposed group to the probability of the event occurring in the nonexposed group. It is typically used in cohort studies and randomized controlled trials, where the incidence of an outcome can be measured directly.

**Rem (Roentgen equivalent man)**: a special unit of any of the quantities expressed as dose equivalent. The dose equivalent in rems is equal to the absorbed dose in rads multiplied by the quality factor (1 rem = 0.01 Sv) [28]. The quality factor for X-rays is 1.

**Sievert**: The special name for the SI unit of equivalent dose, effective dose, and operational dose quantities. The unit is joule per kilogram (J kg^−1^).

**Stochastic effect**: malignant disease and heritable effects for which the probability of an effect occurring, but not its severity, is regarded as a function of dose without threshold [34].

**Tissue reaction**: Injury in populations of cells, characterized by a threshold dose and an increase in the severity of the reaction as the dose is increased further. Also termed tissue reaction. In some cases, tissue reactions are modifiable by postirradiation procedures including biological response modifiers [34]. These were previously called “deterministic effects.”

**Tissue weighting factor**: the dimensionless factor by which equivalent dose is weighted to represent the relative contribution of that tissue or organ to the total radiation detriment resulting from uniform irradiation of the body. The *w*_T_s are judgment values grouped by organs and tissues in the interest of simplicity and rounded to sum to 1.0 [50].

**Training:** applied knowledge and practical aspects of a topic that result in improved efficiency and productivity [16]**.**

**Uncertainty**: lack of sureness or confidence in predictions of models or results of measurements [50].

## Supplementary Information

Below is the link to the electronic supplementary material.

Supplementary file1 (DOCX 1069 KB)

## Data Availability

Not applicable.

## Abbreviations

CIRSE: Cardiovascular and Interventional Society of Europe
*D*: Absorbed dose
*E*: Effective dose
EDE: Effective dose equivalent
FGI: Fluoroscopically guided interventional procedure
Gy: Gray
*H*: Equivalent dose
IAEA: International Atomic Energy Agency
ICRP: International Commission on Radiological Protection
IEC: International Electrotechnical Commission
MPD: Maximum permissible dose
NCRP: National Council on Radiation Protection and Measurements
PET: Positron emission tomography
PPE: Personal protective equipment
R: Roentgen
rem: Roentgen equivalent man
SIR: Society of Interventional Radiology
Sv: Sievert
VR: Virtual reality
*w*_T_: Tissue weighting factor
Z: Atomic number
