# Supplementary material for: Occupational Protection in Interventional Radiology. A Joint Guideline of the Cardiovascular and Interventional Radiological Society of Europe and the Society of Interventional Radiology
Source: Cardiovasc Intervent Radiol. 2026 Feb 12;49(4):716–33. doi: 10.1007/s00270-025-04195-4 (PMC13021820; doi:10.1007/s00270-025-04195-4)
Supplement: Supplementary file 1 — Supplementary file1 (DOCX 1069 KB) [file 270_2025_4195_MOESM1_ESM.docx]

***Annex: Occupational Protection in Interventional Radiology. A Joint Guideline of the Cardiovascular and Interventional Radiological Society of Europe and the Society of Interventional Radiology***

**Abbreviations, Acronyms and Symbols**

Bq Becquerel

CI Confidence interval

CIRSE Cardiovascular and Interventional Radiological Society of Europe

CNS Central nervous system

*D* Absorbed dose

DE Dose equivalent

dĒ Mean energy imparted by ionizing radiation

dm The unit mass of irradiated material

*D*_T,R_ The absorbed dose in a tissue or organ

*E* Effective dose

DMS Dose management system

EDE Effective dose equivalent

EPD Electronic personal dosimeter

ERR Excess relative risk

FGI Fluoroscopically guided interventional procedure

FOV Field of view

Gy Gray

*H* Equivalent dose

*H*_T_ The equivalent dose in a tissue or organ

IAEA International Atomic Energy Agency

ICRP International Commission on Radiological Protection

IEC International Electrotechnical Commission

*K*_a,r_ Cumulative air kerma, also called reference air kerma

kVp Peak kilovoltage

LNT Linear-no-threshold

MPD Maximum permissible dose

NCRP National Council on Radiation Protection and Measurements

OR Odds ratio

OSL Optically stimulated luminescence dosimeter

PET Positron emission tomography

*P*_KA_ Air kerma area product, previously called dose area product

PPE Personal protective equipment

R Roentgen

Rem Roentgen equivalent man

RR Relative risk

SIR Society of Interventional Radiology

Sv Sievert

TLD Thermoluminescent dosimeter

USRT U.S. Radiologic Technologists Study

VR Virtual reality

*w*_R_ Radiation weighting factor

*w*_T_ Tissue weighting factor

**Introduction**

This document accompanies the 2025 joint Guideline on Occupational Protection in Interventional Radiology of the Cardiovascular and Interventional Radiology Society of Europe (CIRSE) and the Society of Interventional Radiology (SIR) [1]. It contains additional information and more detailed reviews of some topics discussed in the Guideline, as well as additional medical physics topics that were not included in the Guideline.

**Radiation Protection Quantities and Units, Personal Dosimetry, and Uncertainty**

**Quantities and Units**

Absorbed dose (*D*) is a measurable radiation quantity defined as the mean energy (dĒ) imparted by ionizing radiation per unit mass of irradiated material (dm) at the point of interest. The unit is J kg^–1^ with the special name gray (Gy) [2]:

*D* is a measurable quantity from which derived quantities can be calculated. It is therefore often the starting point for occupational dosimetry. Numerous assumptions and corrections are applied to the dosimeter measurement to estimate the occupational dose experienced by the wearer.

Once *D* has been determined, two derived radiation quantities can be calculated—Equivalent Dose and Effective Dose. Both are expressed in sieverts (Sv). Multiplying *D* by a radiation weighting factor (*w*_R_) to account for differences in the biological effects of various types of radiation for a given energy deposition yields Equivalent Dose (*H*). For diagnostic energy x-rays, *w*_R_ is 1, so *D* and *H* are the same.

The Equivalent Dose for each tissue or organ (*H*_T_) is the mean absorbed dose in a tissue or organ (*D*_T,R_) weighted by the radiation weighting factor (*w*R) for the type and energy of radiation incident on the body:

*H*_T_ can then be multiplied by a tissue weighting factor (*w*_T_) for that organ or tissue to account for the biological differences in radiosensitivity of various organs and tissues. Summing these values over all organs and tissues allows for an overall estimate of risk, called Effective Dose (*E)*.

Or

*E* and *H* use the most recent weighting factors published by the International Commission on Radiological Protection (ICRP) in Report 103 [3]. Regulations in the U.S. have not incorporated these weighting factors and instead use older factors from ICRP Report 60 [4] that yield slightly different values and have different names: Effective Dose Equivalent (EDE) and Dose Equivalent (DE), that correspond to *E* and *H*, respectively.

**Personal Dosimetry**

A typical personal dosimeter is designed and calibrated to report two operational quantities: Hp(0.07) and Hp(10), where Hp is the personal equivalent dose. These represent the dose equivalent in soft tissue at 0.07 mm and 10 mm depth from the surface of the body, respectively, at the location of the dosimeter. Hp(0.07) from the collar dosimeter worn over protective garments (apron, thyroid shield) provides a reasonable estimate of the dose delivered to the surface of the unshielded skin and to the unshielded lens of the eye. Consultation with a medical physics expert is recommended if the collar dosimeter is used to estimate the dose to the lens of the eye, because the collar dosimeter value does not incorporate the effect of leaded eyewear. In Europe, Hp(10) from the dosimeter worn on the anterior chest inside protective garments is assumed to be a good estimate of the operator’s effective dose (*E*) and was previously considered an adequate indicator of the possible health detriment from radiation exposure. A single under-lead dosimeter does not provide any information about eye lens dose. The formula used to estimate *E* from dosimeter data may be specified by national regulations or by local hospital policy. In the United States, when a protective apron is worn during diagnostic and interventional medical procedures that use fluoroscopy, the National Council on Radiation Protection and Measurements (NCRP) recommends combining the Hp(10) values from both body and collar dosimeters to estimate *E*:

*E* (estimate) = 0.5 HW + 0.025 HN

where HW is the reading from the dosimeter at the waist or on the chest, under the protective apron, and HN is the reading from the dosimeter at the neck, outside the protective apron [5]. However, not all regulatory authorities allow this methodology. (In the U.S., the individual states regulate occupational protection from x-rays). When uncorrected dosimeter readings must be used, they likely substantially overestimate the *E* or EDE of the wearer.

**Uncertainty**

Accurate estimation of occupational radiation dose relies on the proper selection of dosimetry devices, adherence to the requirements and assumptions made by the dosimeter type, appropriate occupational monitoring policies and protocols, and strict compliance with all institutional and regulatory requirements. All discussions of measurement accuracy and uncertainty assume that these requirements are met; without them, uncertainties cannot be accurately estimated and could theoretically be infinite. This underscores the need for robust oversight and a vigilant safety culture to ensure that radiation dose is appropriately monitored.

Uncertainty in the context of occupational dosimetry is complex. There is uncertainty in measurement, as there is with any measured value. This is dependent on many technical factors but is generally considered to be in the 1-5% range [6]. This uncertainty increases as those measured data are then used to estimate organ doses to individuals with a wide range of body habitus, inconsistent measurement locations, and varying irradiation conditions. Because of this uncertainty, the formulas used to derive *E* and EDE intentionally tend toward overestimation. This is desirable for radiation safety purposes but may unduly alarm individuals with high readings whose actual E or EDE is overestimated [5]. The assigned *E* or EDE for any individual must be considered with an understanding that the value is a crude estimate and likely substantially exceeds the actual doses received.

There is also considerable uncertainty in the application of risk estimates for single acute radiation exposures to the occupational situation of chronic exposure to low doses of ionizing radiation. This is not generally accounted for in occupational radiation exposure analyses and is not well understood in the context of occupational exposure.

**Occupational Dosimetry**

**Dosimeters**

Commonly used personal dosimeters include thermoluminescent dosimeters (TLDs), optically stimulated luminescence (OSL) dosimeters, and electronic personal dosimeters (EPDs). TLD and OSL devices are periodically analyzed to determine the dose that they receive. Some EPDs can provide real-time monitoring and display the results while a procedure is in progress. Although many technologies are available, all devices used for regulatory purposes should be appropriately accredited. In fluoroscopically guided interventional procedure (FGI) settings, dosimeters are typically collected and analyzed monthly to provide timely detection of elevated exposure levels, allowing for prompt intervention and mitigation.

Electronic personal dosimeters provide real-time monitoring and instant feedback, allowing for immediate corrective actions if necessary. They may provide audible dose-rate indications and visual displays of dose rates. They can be a valuable tool as part of radiation protection training for both fluoroscopy and CT-guided procedures but are not a substitute for practical radiation protection training [7-9]. EPDs work well in supervised training situations but may be distracting or ignored when routinely deployed. They are typically worn over protective apparel. If they are used for regulatory purposes, they should be accredited for that purpose. In some cases, if a dose management system is available, it is possible to link occupational and patient doses to compare operator practices and optimize occupational radiation protection strategies [10].

The ICRP also recommends the use of an “ambient dosimeter” located on the C-arm (**Figure 1**), to measure the level of scatter dose that would be received if protective apparel were not used [11]. These dosimeters provide backup to personal dosimetry and can be used to assess scatter radiation fields on a continuing basis, demonstrate non-compliance in wearing personal dosimeters, help estimate occupational doses when personal dosimeters have not been worn, integrate occupational and patient protection, and help in optimization of individual radiation protection habits [11-13]. If there is appropriate use of radiation protection tools (e.g., ceiling-suspended shields), measured occupational doses per procedure should be only a few percent of the value measured by the ambient dosimeter [14].

**Fig.1.** The reference (or ambient) dosimeter is located on the C- arm to measure the level of scatter radiation produced in the interventional rooms. This reference dosimeter is attached to the lower part of the C- arm (**arrow**). Well-protected operators should receive much lower doses than these reference dosimeters. Reproduced with permission from [12].


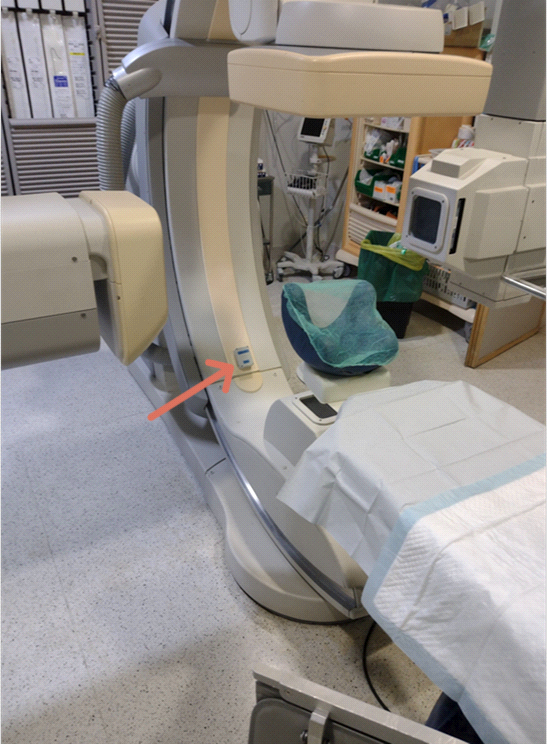


**Dosimeter Use**

The ICRP and NCRP recommend that interventionalists use the two-dosimeter method because the weighted average of the two readings provides a better estimate of whole-body exposure [11, 15]. In 14 of 26 European countries there is a legal requirement to estimate the dose to the lens of the eye [16]. The dose to the lens of the eye may be estimated from the collar dosimeter. The two-dosimeter method also has the advantage that the waist badge can be used to monitor fetal dose during pregnancy. This method is common practice in Europe, where it is frequently required by national regulation. In some countries, monitoring of hand dose is mandatory.

Both the single dosimeter and two-dosimeter methods have disadvantages. If the single dosimeter is worn over the apron, it is not possible to estimate whole body exposure because there is no way to determine the protective effect of the apron, or if an apron is even worn. If the dosimeter is worn under the apron, as is the practice in some countries, the dosimeter cannot be used to estimate the dose to the lens of the eye. With the two-dosimeter method, if the dosimeters are switched, the whole-body value can be overestimated by up to a factor of 10. When worn correctly, the two-dosimeter method may overestimate whole-body dose for U.S. workers by nearly a factor of 3 due to the formula used to calculate EDE [17]. The two-dosimeter method is also more expensive. In the United States most states require only a single dosimeter [18], so the two-dosimeter method is less commonly used, primarily because staff often wear their dosimeters incorrectly [17, 19, 20]. Use of the two-dosimeter method in the U.S. has declined over time [17].

**Evaluation of Personal Dosimetry Data**

Personal dose records should contain the information required by regulation and the dose values measured by all personal dosimeters used by individuals involved in interventional radiology. They should be reviewed regularly by a medical physics expert or radiation protection expert, with results communicated to users as required by regulation or facility policies. Dose values should be reviewed as required by local regulations (in the U.S., at least quarterly) with results reported to individuals at least annually. Aggregate dosimetry results can be reported to department leaders who should be familiar with the workloads of the badged personnel.

Review of dosimetry records should ensure that annual dose limits are not exceeded and that reported values are consistent with those expected from that worker’s duties. Abnormally high or low values should be investigated, even if the user is unlikely to exceed annual exposure limits. Normalization of occupational doses by number of cases or by metrics such as total *P*_KA_ or total *K*_a,r_ may be possible, and is useful for comparing occupational exposure between cohorts with varying caseloads.

The ICRP recommends that “There is no need to wait until an annual dose limit or constraint has been exceeded to become aware that protection was not optimized. Non-optimized protection can be detected by establishing an investigation level” [11]. Because badge readings for interventional operators are expected to be higher than for most other hospital workers, their investigation levels should be set to avoid non-productive investigations of otherwise expected exposure levels. ICRP publication 103 recommends against setting triggers as arbitrary fractions of the dose limit [3]. The World Health Organization recommends investigation when monthly exposure reaches 0.5 mSv for effective dose, 5 mSv for dose to the lens of the eye, or 15 mSv to the hands or extremities [21].

Typical staff dose readings for different types of procedures have been published in the literature. Depending on the type of procedure and the technique used, median operator effective dose, per procedure, ranges from 1.6 to 33 µSv [22, 23]. As noted in the guideline, a busy interventional radiologist who takes all appropriate radiation safety precautions is unlikely to exceed the 20 mSv ICRP limit for annual exposure and more likely to have an *E* of 2–6 mSv/y [11, 24, 25].

When professionals have very low doses in comparison with other staff doing similar work, proper use (or lack of use) of personal dosimeters should be investigated. Abnormally high doses should also be evaluated. Investigation of abnormally high or low personal dose value begins with a check of the validity of the dosimeter reading. Potential sources of invalid dosimeter readings include not wearing dosimeters at all, improper badge positioning (including mixing up over- and under-apron badges), and storage of badges on protective equipment such as aprons or thyroid collars within the fluoroscopy suite.

Sources of valid but elevated readings include increased operator caseload, fluoroscope protocol modification, initiation of new, more complex procedures, and poor radiation safety practices such as failure to utilize available shielding or inappropriate use of high dose protocols or modes of operation. Operator caseloads can often be extracted from dose monitoring software or electronic medical records for ready comparison. If the abnormal doses are observed for several professionals, the results of the quality control analysis of the X-ray system should also be investigated. If an invalid reading is suspected, the reading for the individual’s next monitoring period should be reviewed to ensure the problem has been corrected. If it has, the invalid reading can be replaced with an average monthly exposure in the dose record.

When a seemingly valid elevated reading is encountered, the worker should be notified and asked if there was a change in work habits that could explain the increase in radiation exposure. Was a new type of procedure initiated during the monitoring period? Were procedure techniques or equipment settings modified? If so, did these new methods require remaining in closer proximity to the patient or increased patient dose? Did procedure workload or complexity increase? Sometimes, a temporary cause is found. If this is the case, dose levels should return to usual levels during the next monitoring period, when workload returns to normal, equipment settings are corrected, or there is additional experience with a new procedure or technique. The individual’s dose reading for the next monitoring period should be reviewed to confirm that dose levels have returned to the expected range.

For legitimately high dose readings with no obvious cause, operator working habits may be observed by a medical physics expert or interventional physician with knowledge of radiation protection principles and the operation of the specific equipment being used. Attention should be paid to equipment settings such as pulse rate and mode of operation, the worker’s proximity to the patient, the use of collimation, and the use of equipment-mounted shields and protective equipment. When available, real-time dosimeters are useful for visualizing the radiation protection effects of these elements of practice. With adequate cooperation and attention to dose reduction principles, forced limitation of workload to ensure compliance with dose limit is generally not needed.

**Radiation Effects**

The earliest experimenters with x-rays shielded neither the x-ray tube nor themselves. Some developed cancer as a result [26, 27]. Others developed chronic dermatitis [28]. Radiologists practicing in the 1940s and 1950s could receive occupational radiation doses far higher than those permitted today [29]. In 1936, the recommendation for a permissible whole body occupational exposure was 0.1 R/day (1 mSv/day) [30]. It decreased in the U.S. to the current occupational limit of 50 mSv/y (5 rem/y) only in 1958 [28]. The ICRP established a recommended whole body occupational dose limit of 20 mSv/y, averaged over 5 years, in Publication 60, published in 1991 [4].

Much of what we know about the risk of radiation exposure in humans comes from epidemiologic studies. Analysis of risk per unit organ dose (“dose-response”) is the preferred modeling approach used in these studies [31], with results reported in terms of the excess relative risk (ERR) per unit dose (e.g., Gy or Sv). Organ doses are more appropriate for use in dose-response assessment than effective doses because they are more biologically meaningful. Dose-response analyses are more statistically powerful than those that use categorical or binary comparisons (e.g., relative risks (RRs) or odds ratios (ORs) comparing exposed versus unexposed) because they use data from the entire dose range and are less prone to exposure misclassification and confounding. Dose-response analyses are also preferred to an approach that relies on surrogate exposures (e.g., number of times exposed, distance from radiation source, number of years worked, number of medical procedures performed).

Organ dose reconstruction for radiation epidemiologic studies has become increasingly sophisticated, but uncertainties around organ dose estimates can be quite large and can hinder the interpretation of epidemiologic studies. Uncertainties are not necessarily higher at lower doses, but they can make it more difficult to “detect” a significant association at low doses [32, 33]. Furthermore, in a cohort study setting, random errors in estimates of dose tend to drive an association toward null (no effect) rather than to artificially inflate, or induce, an association [32]. Therefore, it is unlikely that inaccuracies in dose reconstruction can “explain away” a statistically significant positive dose-response association.

Findings from epidemiologic studies of occupationally exposed workers are described below. There are important considerations when interpreting these findings. First, occupationally exposed workers tend to be healthier, on average, than the general population and have lower age-adjusted mortality rates. To minimize such biases, the most valid assessments of risk in cohort studies tend to be those conducted using internal comparisons (i.e., among the study subjects rather than with an external group such as the general population) with statistical adjustment for potential confounding factors, such as cigarette smoking. Second, the absolute risks (i.e., number of excess events) of cancer, circulatory, and neurological outcomes attributable to occupational radiation exposure are estimated to be very small. For instance, a survey of medical workers in interventional radiology departments in South Korea estimated a lifetime excess risk of cancer attributable to occupational radiation exposure of 338, 121, and 156 per 100,000 male radiologists, male radiologic radiographers, and female nurses, respectively [34]. On the other hand, estimates of relative risk (as described below) may be substantially underestimated, thus yielding an underestimate of the absolute risk. Third, results of studies of monitored workers generally do not account for lack of compliance in the use of dosimeters. Unfortunately, this is common, particularly among interventionalists [17, 25, 35-37], and would lead to attenuation in the estimated risks. Also, medical worker cohorts generally report high and consistent use of lead aprons and room shields, particularly in interventional radiology departments [38]. In settings where the use of personal protective equipment is low, the risks of cancer and other radiation-associated outcomes may be much higher.

**Cancer Risks**

Ionizing radiation is one of the most well-studied carcinogens and is an established cause of nearly all types of cancer [31]. In epidemiologic studies with high-quality organ dose reconstruction, risks of most type of cancer tend to increase linearly across the full range of dose [39], with no clear evidence to date of a dose threshold (i.e., level of dose below which there is no risk) [40]. This is the basis for adoption of the “linear-no-threshold” (LNT) model by national and international scientific committees as the most pragmatic and prudent model for radiation protection purposes [3, 41-43].

Our current understanding of cancer risks associated with protracted (repeated) low-dose radiation exposures has been derived primarily from studies of acute (one-time) exposures. The Life Span Study includes about 120,000 Japanese atomic bomb survivors identified 5-10 years after the 1945 bombings of Hiroshima and Nagasaki. It is widely regarded as the “gold standard” of radiation epidemiology research and has been the source of many comprehensive reports of radiation-related risks of total and specific types of solid cancers and hematologic malignancies [44]. Results from this study indicate that adult radiation exposures are most strongly associated with cancers of the bladder, female breast, lung, and brain and central nervous system, as well as leukemia (excluding chronic lymphocytic leukemia) [31].

In recent years, large cohort studies and pooled analyses of occupationally exposed medical and nuclear workers and patients undergoing imaging procedures have contributed more direct information about the health effects of repeated (protracted) exposure to low-dose radiation. In general, these studies have not found much of a difference in risks of cancer (and some non-cancer outcomes) between cumulative dose spread out over time versus the same dose received all at once [45-49].

In the U.S., it is expected that approximately 40% of all individuals will develop cancer at some point in their life, and 20% of individuals will have cancer as their cause of death, as shown in **Table 1** [50]. In a large multinational cohort study of nuclear workers monitored with personal dosimeters, higher occupational organ/tissue-absorbed radiation dose was associated with a modest increased risk of death due to solid cancer overall and lung cancer specifically [49], as well as a relatively stronger increase in risk of death due to leukemia (not including chronic lymphocytic leukemia) [47]. In the U.S. Radiologic Technologists Study (USRT), occupational organ/tissue-absorbed radiation dose, derived primarily from objective badge records, was positively associated with female breast cancer incidence and mortality and lung cancer mortality (particularly in radiographers with <20 pack-years of smoking history) [51, 52]. However, no clear dose-response associations were observed for leukemia or cancers of the skin, brain/central nervous system, or thyroid [53-56]. Similar null dose-dependent findings for brain/central nervous system cancer, thyroid cancer, and leukemia were observed in the French and South Korean medical worker cohorts, although the follow-up duration and number of events in these cohorts were much more limited compared to the USRT cohort [57-59].

Overall cancer mortality rates in a large cohort of U.S. male physicians who specialized in interventional radiology, interventional cardiology, or interventional neuroradiology were significantly lower compared to rates in an unexposed comparison group (psychiatrists). A non-significant increased rate of leukemia mortality was observed among interventionalists who graduated from medical school before 1940, who likely received the highest lifetime exposures [60]. The number of female physicians was too small to draw any conclusions about mortality risks for this group, so an increased risk of death from female-specific cancers, including female breast cancer, could not be excluded. Also, the follow-up of this cohort was relatively short, and the average age of the physicians at the end of follow-up was only 51 years, so additional follow-up will be required. A recent case-control study found no association between the number of ionizing radiation procedures performed and the risk of cancer among physicians in Ontario, Canada, over a 30-year period (1991-2021) [61]. Results from selected large worker cohorts are shown in **Table 2.**

Case series have raised concerns about the risks of brain and central nervous system tumors in physicians who perform FGI procedures, including a higher frequency of left-sided versus right-sided brain tumors, consistent with generally higher occupational exposure on the left side [62, 63]. Such reports are difficult to interpret due to the lack of an unexposed comparison group. While there is a lack of convincing evidence linking occupational radiation exposure and malignant brain/CNS tumors, an elevated risk for benign brain/CNS tumors would be consistent with findings from the Japanese Life Span Study and thus cannot be ruled out [31]. Estimates of radiation-associated risks of benign brain/CNS tumors from occupational cohort studies are forthcoming.

**Lens Opacities**

Radiation-induced cataracts have been observed in health care workers, including those who participate in FGI procedures, as well as several other radiation-exposed populations [64-74]. A systematic review of the health effects of occupational exposure to ionizing radiation found that the occurrence of lens opacities among interventionalists varied significantly, from 16% to 47% [75]. Results from selected large worker cohorts are shown in **Table 3**. In general, posterior subcapsular and cortical cataracts have been more consistently linked with radiation exposure, although there is some limited evidence of an association with the nuclear type. The large USRT cohort demonstrated a linear dose-dependent relationship between occupational radiation exposure and self-reported diagnosis of cataract, with a significant increased risk at radiation doses to the lens of the eye <100 mGy and no evidence of a threshold (i.e., dose below which there is no risk) [74]. A positive but non-significant association was observed after the analysis was restricted to surgically removed cataracts. In the same cohort, radiographers who reported regularly assisting with FGI procedures had an 18% higher risk of cataract during the follow-up period compared to those who did not. This risk increased with the frequency of procedures performed, particularly those performed while standing within three feet (1 m) of the patient [65], and was only observed among radiographers who did not consistently (>50% of the time) use room shields or lead glasses. This cohort was also studied for the risk of glaucoma and macular degeneration [76].

Several relatively small studies compared the prevalence of lens opacities based on a clinical eye examination, use of personal protective equipment, and sometimes occupational doses, between interventional cardiologists and an unexposed physician comparison group. A French study of 106 interventional cardiologists and 99 unexposed physicians found no difference in the prevalence of lens opacities overall, with a slightly higher proportion of posterior subcapsular lens opacities in the exposed group [66]. The risk increased with duration of activity but not workload and appeared to be lower with regular use of lead glasses. In a Finnish study of 21 radiation-exposed and 16 unexposed physicians, cumulative occupational exposure was not clearly associated with lens opacities of any type, based on ophthalmological examinations, nor were differences observed for the left versus right eye [67]. A similar study conducted in Germany compared interventional cardiologists and unexposed physicians, with a higher proportion of lens opacifications observed in the exposed group [68]. Interestingly, 68% of the participating interventional cardiologists reported never wearing lead glasses, while about 80% reported using ceiling-suspended and table shields and >90% reported using a lead apron and/or thyroid collar. In a study of 116 South American interventional cardiologists, nurses, and radiographers, the cardiologists had a 3.2-fold higher prevalence of posterior lens opacification compared to 93 age-matched unexposed individuals, while nurses and radiographers had 1.7-fold higher prevalence [69]. Cumulative median values of lens doses were estimated at 6.0 Sv for cardiologists and 1.5 Sv for nurses and radiographers. The small sample size and low participation rates in these and similar studies conducted elsewhere make it difficult to draw firm conclusions. However, they are generally consistent in demonstrating evidence of radiation-induced lens opacities [70-72].

Between 2011 and 2014, a survey was administered to attendees of annual Italian interventional cardiology and electrophysiology conferences and at a local research facility, capturing medical history, work history, and lifestyle-related factors [73]. The study found that workers, particularly physicians, performing fluoroscopically guided cardiovascular procedures had a higher prevalence of several potentially radiation-related health issues, including cataracts, cancer, orthopedic issues, and anxiety/depression, than unexposed subjects.

**Cardiac and Neurologic Outcomes**

The effects of high-dose radiation exposures on cardiac and neurologic outcomes (including cognitive function) have been demonstrated clearly in experimental and epidemiologic studies. There is increasing evidence that links repeated low-dose radiation exposure with cardiac and neurologic tissue reactions [48, 77]. A small number of studies have suggested lower memory and olfactory performance, higher level of oxidative stress, and reduced glutathione in interventional cardiology versus unexposed medical staff [62]. The risk estimates for cardiac and neurologic outcomes vary, likely because the risk of a cardiac or neurologic outcome for a given absorbed dose is potentially less than that for cancer or cataract development. Also, risk estimates for cardiac and neurologic outcomes are more susceptible to confounding and the other types of bias inherent in observational cohort studies.

The results of analyses of large, occupationally exposed radiation cohorts for the risks of cardiovascular and neurologic outcomes are summarized in **Table 3** [60, 78-82]. In the large INWORKS cohort, which includes >300,000 nuclear workers monitored with personal dosimeters, a significant positive association was observed for cumulative radiation organ/tissue absorbed dose and circulatory disease mortality [78]. In a cohort of >50,000 South Korean male diagnostic medical radiation workers, positive linear dose-response associations for cardiovascular disease, ischemic heart disease, and cerebrovascular disease were observed, based on organ/tissue absorbed doses derived from personal dosimeter readings. These estimates were not significant but were based on a relatively short follow-up period and a much smaller number of mortality events than the INWORKS study [79]. Dose-response estimates from the USRT cohort of radiologic technologists (who, like the INWORKS and South Korean cohort members, were also monitored with personal dosimeters) on radiation exposure and circulatory disease have not yet been published. However, after adjusting for age, birth year, gender, race, smoking history, and body mass index, analysis of the USRT cohort has shown that subjects who began working as radiographers in earlier calendar years, and those who worked as radiographers for more than five years before 1950 (when permitted occupational exposures were much higher than today) had a higher risk of death from ischemic heart disease and cerebrovascular disease than those who began working more recently [83]. Radiographers who reported working at least monthly with FGI procedures experienced a higher incidence of cerebrovascular disease compared to those who never worked with these procedures [84].

A large cohort study of U.S. male physicians who specialized in interventional radiology, interventional cardiology, or interventional neuroradiology found lower rates of death from cardiovascular and neurological and mental causes compared to unexposed physicians [60]. The number of exposed female physicians was too small to draw any definitive conclusions. The follow-up of this cohort was relatively short, and the average age of the physicians at the end of follow-up was only 51 years; additional follow-up will be required.

A meta-analysis of six occupational groups (totaling 517,608 workers) within the Million Person Study, comprising about 50% of the cohort, found an overall positive association between estimated cumulative radiation dose to the brain and death due to Parkinson’s disease [80]. This finding is consistent with a study of workers in a Russian nuclear production facility [81]. However, in a French nuclear worker study, no association was observed for Parkinson’s disease mortality, but occupational exposure was significantly positively associated with dementia/Alzheimer’s disease mortality [82].

**Musculoskeletal Injuries and Prevention**

In a survey of SIR members, musculoskeletal symptoms were reported by 88% of respondents. Fifty-eight per cent of those reporting these symptoms ascribed them to work-related activities [85]. Other surveys also demonstrate that large numbers of interventional radiologists (and interventional cardiologists and surgeons) report neck, thoracic or back pain [86-88]. Compared to the general population, interventional radiologists exhibit 1-year prevalence rates at the high end of the range for shoulder and low back symptoms and double the mean for neck symptoms [85]. These symptoms may result in time lost from work, leaves of absence, practice restriction, burnout, and early retirement [86, 89, 90].

Some individuals are particularly susceptible to developing musculoskeletal injuries. In a survey of staff who work in interventional radiology and interventional cardiology units at Mayo Clinic affiliated hospitals, Orme et al. found that factors associated with musculoskeletal pain in these workers were female sex, increasing time participating in interventional procedures, and increasing time wearing lead aprons [91]. In a survey of SIR members, Morrison et al. identified female gender, above-normal body mass index, and a practice length of 10 years or more as factors associated with a higher risk of moderate-to-severe symptoms [85]. In a survey of medical radiation workers in China, Li et al. identified female gender, alcohol consumption, repetitive motions, working overtime, and prolonged maintenance of the same posture as risk factors for neck, shoulder, and back work-related musculoskeletal injuries [92]. They also identified specific risk factors associated with back pain among interventional radiologists: repetitive motion, inadequate recovery time, prolonged standing, axial loading on the spine, and awkward postures. Female gender is also associated with a higher frequency of musculoskeletal symptoms in surgeons, especially neck and upper extremity pain [86].

Posture is defined as the spatial arrangements of body parts as they align to perform a task [87]. Incorrect posture can lead to fatigue, discomfort, stiffness, and numbness in the back, neck, shoulder, and legs, as well as long-term disability or the need for corrective surgery [93]. The body should be kept in a neutral stance to alleviate tension in the torso and extremities. Procedure rooms should be designed to foster proper ergonomic positioning of the equipment with respect to the operator and patient to decrease the risk of posture-related and repetitive-stress injuries [86, 89, 94-96]. Fluoroscopes and other imaging equipment should be positioned to allow the operator to stand comfortably, without bending, leaning, or reaching.

Table height should be adjusted so that the operator does not need to bend over and so that the elbows can be held in a neutral position [97]. Positioning the table properly allows the biceps muscle to remain at less than 15% of maximum muscle activity while reducing back, shoulder, and wrist discomfort [89, 98]. Inappropriate table height can also cause sustained shoulder abduction, which results in deltoid muscle fatigue and deterioration in the performance of tasks requiring high accuracy [99]. It is important to avoid excessive or sustained shoulder abduction and internal rotation, a position that requires the greatest workload from the deltoid and trapezius muscles [87].

The monitor that displays fluoroscopy and reference images and physiologic data should be in front of the operator and just below eye level, to avoid unnecessary extension and rotation of the cervical spine [87, 89, 93, 94]. The fluoroscopy suite should be designed so that this monitor position is possible for all the positions around the procedure table where the operator is likely to work, including the patient’s neck, arm, abdomen, groin, and foot. This may require additional monitors.

The fluoroscopic monitor’s distance from the operator should be optimized. The goal is an upright posture with the spine in neutral position and without any forward tilt [87]. Viewing distance affects the eye’s ability to extract details from an image. The optimum viewing distance depends on the combination of field-of-view (FOV), the size of the monitor, the area of the monitor occupied by the image and the monitor pixel size [100]. For a fixed FOV, optimum viewing distance increases with increased monitor size. Interventionalists often lean over the patient to improve their ability to see detail in displayed images (**Figure 2**). With larger monitors, operators can stand further from the monitor and are more likely to assume a neutral spine position.

**Fig.2**. **Left.** With the monitor positioned appropriately (vertical white rectangle), the operator can see detail in the fluoroscopic image while maintaining a neutral spine position. Note also that table height is appropriate, so that proper arm and elbow position are achieved. **Center.** With a smaller monitor or smaller image, the monitor must be moved closer to the operator for details in the displayed image to be appreciated. If this is not done, operators are more likely to lean over the patient to improve their ability to see image detail. **Right.** Even with a large monitor and a large image, if the monitor is placed too far from the operator, proper posture is not maintained.


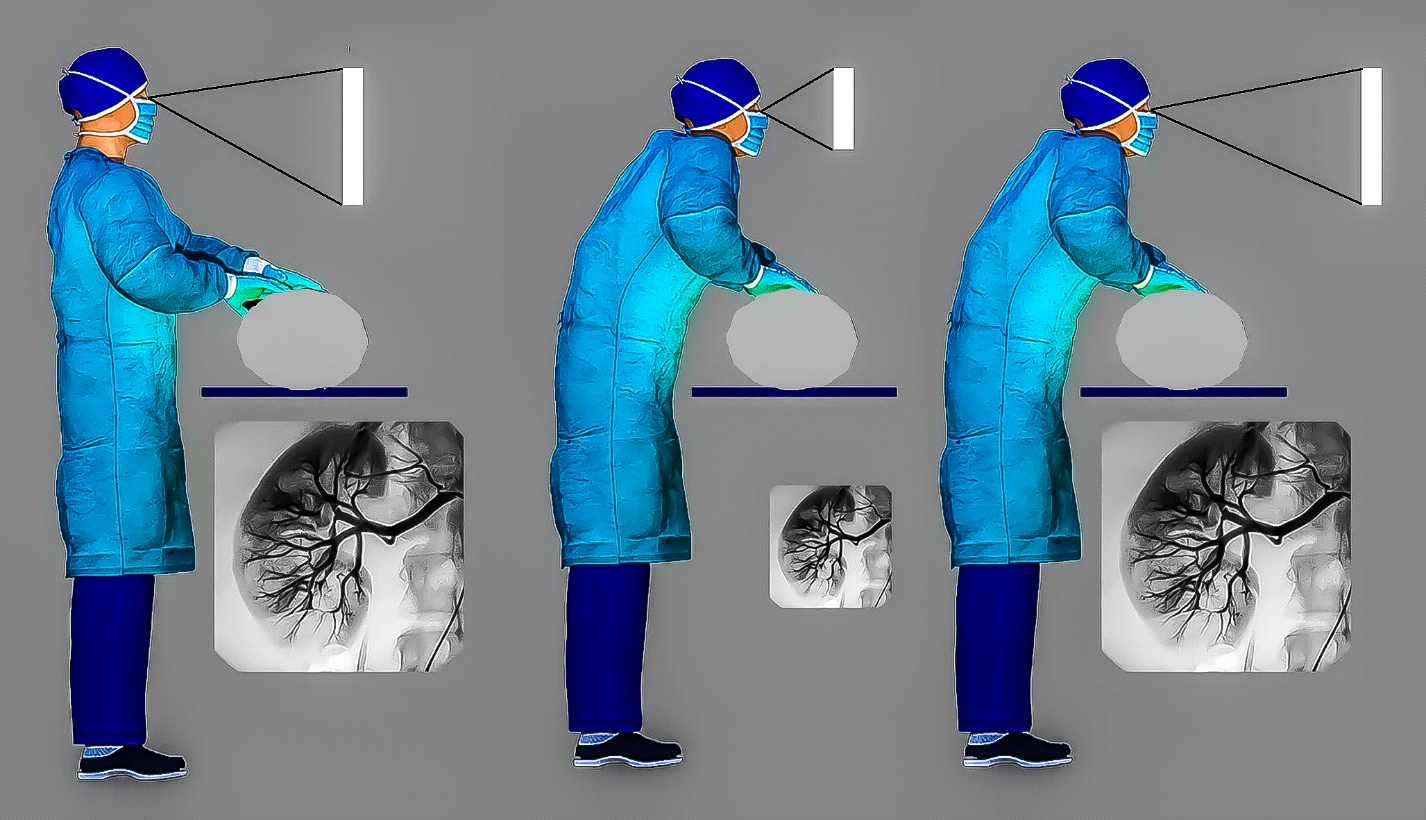


Spending long periods of time in a constant position, particularly an awkward position, should be avoided [87, 89]. Taking a break between cases can interrupt the cycle of pain [94]. Taking 1-2 min “microbreaks” every 20 to 60 min during a case has been shown to improve mental focus and physical performance and should be considered [86, 87, 89, 93]. Recommended stretching exercises can be performed during these breaks [93]. Hallbeck et al. [101] studied the effect of microbreaks with exercises in a group of 56 surgeons. The standardized exercises, described in the paper, took 1.5 to 2 min to perform and focused on the neck, back, shoulders, hands, and lower extremities. Of the 56 surgeons, 57% reported improved physical performance with use of microbreaks, 43% reported no change, and none reported decreased performance. There was no significant change in operative time. Impact on operative flow was minimal. At the conclusion of the study, 87% of participants wanted to incorporate microbreaks with exercises into their operative routine [101]. Park et al. [102] reported very similar results but may have been studying the same group of surgeons.

While most attention has been paid to ergonomic issues in the fluoroscopy suite, musculoskeletal injuries may also result from the awkward positions that can be necessary to perform CT, ultrasound-guided, and in-bore MRI-guided interventions [89, 103]. During CT interventions, the operator must often lean over the patient or bend to perform procedures, especially when CT fluoroscopy is used. The CT monitor in the room should be positioned to minimize unnecessary extension and rotation of the cervical spine. During ultrasound guided procedures, the operator may be forced into an awkward position of the trunk, neck, and upper extremities while maintaining a firm grip on the ultrasound transducer. Axial twisting and excessive reach during these procedures can lead to back, neck, and shoulder pain. To avoid these issues, arm abduction should be limited and neutral body positioning maintained by positioning the ultrasound monitor in front of the operator whenever possible.

Treatment of musculoskeletal injuries is multidisciplinary. The most important intervention, rest, may be very difficult to achieve [94]. Eliminating or changing the activity that causes the injury is ideally the first step in treatment. Medication—nonsteroidal anti-inflammatory agents, paracetamol (acetaminophen in the U.S.), muscle relaxants, or oral analgesic agents—is frequently indicated. Therapeutic massage, stretching exercises, physical therapy, yoga and acupuncture may all play a role [86, 87, 94]. If a specific underlying anatomic cause for the pain is identified, steroid injections, blocks, nerve root ablation or surgery may provide benefit.

Injury prevention should be the goal. Designing the interventional facility in accordance with ergonomic principles, as described above, is basic to prevention, as is minimizing or eliminating those practices that predispose to injury. An additional approach is prevention through conditioning [86, 87, 89, 94]. Chang et al. [104] reviewed studies of core strength training for patients with chronic low back pain. They observed that core strength training is more effective than typical resistance training with machines or free weights for alleviating chronic low back pain and recommended focusing on training of the deep trunk muscles. Valenza et al. [105] studied 54 patients with chronic low back pain, randomly assigned to an 8-week Pilates exercise program or to receiving written information only. At the end of the study, the Pilates group had significantly less pain and disability and significantly better flexibility and balance. Yoga has been reported to promote both physical and psychological health, and to treat low back pain [87, 94]. Kumar et al. [106] studied the effect of Isha Hatha yoga in 102 healthy individuals who underwent an intensive 21-day training program, of whom 76 were evaluable. Isha Hatha yoga stresses the physical components of yoga and aims to achieve overall fitness through unique practices and postures. The training resulted in significant improvement in core stability and standing balance. Physical therapy can be used with techniques to foster spinal and extremity movements, limb stretching, and joint mobilization, as well as stabilization exercises with progression to strength, functional, and aerobic training [87]. However, before adopting any conditioning program, consultation with an appropriate therapist is recommended, and any such program should be performed in a stepwise fashion [87].

**Radiation Protection Tools**

**Personal Protective Equipment**

For physicians and staff who perform interventional radiology procedures, radiation protection typically requires wearing a protective apron. Although devices exist for providing suitable radiation protection without the need to wear an apron (freestanding, suspended, and mobile shields), they are expensive and often not suitable for every procedure an interventional radiologist needs to perform [107]. Anything that interferes with necessary procedure performance, thereby lengthening the procedure and increasing the dose to the patient and staff, should be avoided [11]. Apron wear contributes to the risk of musculoskeletal injury [108]. Aprons should be chosen carefully and must fit properly [11, 89, 94, 109, 110]. The risk of musculoskeletal injuries due to the apron’s weight should also be considered in the optimization of protection [11, 108]. Unless national or local regulations specify a minimum lead or lead equivalent thickness [18], an appropriate thickness should be based on the wearer’s under-apron dosimeter readings [11, 15]. The label on the apron is not necessarily a reliable guide to the degree of radiation protection provided [111].

Apron construction is important in determining the distribution of the apron’s weight on the shoulders and back. Two-piece aprons (vest and skirt) place less weight on the shoulders than do one-piece aprons worn with a belt; a one-piece apron worn with a belt places less weight on the shoulders than a one-piece apron without a belt [110]. Unfortunately, while some apron designs may be helpful to reduce injury to the upper extremities, none of them reduce the weight supported by the hips.

Aprons do not completely protect the lateral chest wall and axilla due to the presence of arm holes. The addition of a lead-equivalent sleeve significantly reduces radiation dose to this portion of the chest and axilla when compared with standard leaded aprons alone in both clinical and simulated settings [112, 113]. These sleeves may be useful to reduce radiation exposure to the upper outer quadrant of the breast [114, 115].

Leaded eyewear reduces eye radiation exposure substantially. A review of the effectiveness of leaded eyewear showed that the shielding effect ranges from 10% to 90% depending on eyewear design and lead equivalence [116]. In particular, minimizing the gap between the inferior portion of the lens and the wearer’s skin is important [117]. The orientation of the wearer’s head, complexity of the procedure, and wearer movement also affect the extent of shielding.

**Shields**

The walls, windows, and doors of fluoroscopy rooms include structural shielding to reduce radiation levels in surrounding areas to acceptable levels. Staff should perform their duties from a shielded control room whenever possible. To avoid interrupting a critical procedure, doors into the fluoroscopic room should not be interlocked. This means that staff should monitor these doors and keep them closed as much as possible.

Fluoroscopes are typically supplied with flexible protective drapes hanging below the table, extensions above the table, and large ceiling-mounted transparent shields. These shields have the potential to provide more protection than PPE alone while not adding weight to the user. Suspended shields are typically 0.5 mm nominal lead-equivalent thickness. The effectiveness of ceiling-suspended shields is closely linked to their proximity to the patient – the closer to the patient, the larger the shadow created by the shield. Ceiling-suspended shields provide protection for the entire head and neck but cannot always be placed to provide effective protection. Even when they are used, protective eyewear should still be worn [118]. All of these shields should be installed and used whenever possible, as proper use can reduce the scatter to the operator by up to 90% [11, 119]. Protection for non-operator staff may be less.

Radioprotective drapes can be placed directly on the patient. These include both sterile single-use and reusable (placed in a sterile bag) versions. Staff must be in their shadow if they are to be effective. Care must be taken with their placement. If they enter the FOV, they will affect tube output and increase patient dose.

Other types of shielding systems are available that are intended to allow some staff to potentially forego traditional PPE. These types of shields typically provide 0.5 – 1.0 mm lead equivalence. Free-standing mobile shields have long been used to protect non-operator staff such as nurses and anesthesia personnel. Careful placement of mobile shields between the patient and staff can create effective radiation shadows, protecting most, if not all, of the body of those in the shadow. There are also shielding systems that resemble traditional PPE and are designed to protect the operator without the added weight of PPE [107, 120, 121]. Some of these are mounted to either the ceiling or a floor-mounted movable gantry [107]. There is some evidence that their use may reduce ergonomic posture risk to the torso compared to conventional lead aprons [122].

There are large, free-standing barrier systems with multiple components that maintain access to the patient while shielding surrounding areas [123-126]. These devices can be heavy and may not be mechanically compatible with fluoroscopic hardware. They can be obtrusive and may limit movement within the fluoroscopy suite. They may limit gantry and table motion and can impede procedural performance if the patient table must be panned during the procedure or steep gantry angles are required. Patient communication and access may also be impeded. Policies are needed to manage these devices in the event of a medical emergency. Barrier systems are also available that are placed on the table-top or gantry [127, 128]. Practical issues include reducing the maximum weight of a patient allowed on the table or unbalancing the fluoroscopic C-arm.

**Shield Use**

Free standing mobile shields should be rolled into an appropriate position when needed to supplement table-mounted shielding. For example, they should be used to protect staff when they are working near the patient’s head, feet, or other areas outside of the shadows of other radiation protective devices. Versions are available that are intended to provide an additional layer of operator protection where necessary.

Large free-standing barrier systems can be moved into place at the start of a procedure. It is not practical to move them routinely during the procedure. Adequate attention must be paid to minimizing gaps in their radiation shadow throughout the procedure. These barrier systems may provide a radiation shadow sufficient to allow operators to work without PPE. While they can remove the need for personal PPE for the operator for some procedures, they generally only protect a limited number of staff on the operator’s side [126]. Others in the room still require PPE.

Depending on local badging requirements, occupational monitoring may need to be modified to account for the use of protective devices that allow staff to perform procedures without wearing PPE. Occupational dosimetry data can be useful to study the effectiveness of the radiation protection strategies in use and to further optimize them.

**Practical Advice to Reduce or Minimize Occupational Dose**

In both fluoroscopy and CT fluoroscopy, decreasing patient dose will result in a proportional decrease in scatter dose to the operator [11]. Therefore, techniques that reduce patient dose will generally also reduce occupational dose. This is a “win-win” situation: operators, staff, and the patient all benefit. For any intervention, review existing imaging studies to define the relevant anatomy and pathology and to plan the interventional procedure. Good procedure planning prior to the intervention will help reduce procedure time and radiation dose.

Operators and all staff involved in the procedure should have a general knowledge of safe operating practices in a radiation environment. Operators should be thoroughly familiar with the operation of each fluoroscope and CT scanner that they use. If appropriate medical simulators are available, operators and trainees should consider using them to learn and practice new skills before applying them to patients.

All interventional radiology operators and staff need to know their own occupational dose to ensure that they are working safely. The only way to know this is to wear one’s dosimeter(s). Occupational dose information will not be accurate unless dosimeters are always worn and worn correctly.

**Fluoroscopically Guided Procedures**

Specific techniques can be used with fluoroscopically guided procedures to reduce occupational dose. The recommendations given here are largely unchanged from those in the previous version of this guideline [129].

*Minimize fluoroscopy time*. Fluoroscopy should be used only to observe objects or structures in motion. Review the last-image-hold image or fluoroscopy loop for study, consultation or education instead of using additional fluoroscopic exposure. If available, record a fluoroscopy loop to review dynamic processes. Use short taps of fluoroscopy instead of continuous operation. Fluoroscopy to determine or adjust collimator blade positioning can be eliminated by using the virtual collimation feature, when present, in conjunction with a last-image-hold or fluoroscopy loop image.

*Minimize the number of radiographic images*. For digital subtraction angiography, use variable frame rates tailored to the examination (e.g., 1 image/sec for 6 seconds, then 1 image every other second for 24 seconds for arteriography of the celiac axis) instead of a constant frame rate (e.g., 2 images/second for 30 seconds). Tables of suggested imaging sequences were available in some older standard interventional radiology textbooks but are absent in most current textbooks. For documentation, use stored last-image-hold images instead of acquiring radiographic images if the last-image-hold images provide acceptable image quality. When available, use a stored fluoroscopy loop instead of a radiographic acquisition (digital subtraction angiography) if the image quality is adequate to document the findings.

*Use the lowest dose settings* that provide adequate image quality together with all available patient dose reduction technologies. These include low fluoroscopy dose-rate settings, low frame-rate pulsed fluoroscopy, removal of the anti-scatter grid for infants, small children, and small body parts in adults [130-132], spectral beam filtration, and use of increased x-ray beam energy. Catheters with highly radiopaque tips are easier to see. Improved image processing within the fluoroscopic unit can compensate to a considerable degree for the reduced image quality due to decreased exposure levels. This technique reduces dose at the cost of somewhat decreased image quality.

*Use good imaging-chain geometry*. Position the patient table at a comfortable height for the operator unless patient peak skin dose is a concern, in which case the distance from the patient to the X-ray tube should be maximized. Place the image receptor as close as possible to the patient.

*Collimate*. Adjust collimator blades tightly to the area of interest. Tight collimation reduces patient dose and improves image quality by reducing scatter. When beginning a case, position the C-arm over the area of interest, with the collimators almost closed. Open the collimators gradually until the desired FOV is obtained. When possible, position the collimator blades without fluoroscopy by using virtual collimation.

*Operators and staff* *should position themselves in a low scatter area* *and stay as far away from the x-ray beam as possible*. Operators should use tubing extensions or needle holders so that their hands are away from the exposed field. Operators should never place their hands in the x-ray beam. Use power injectors for contrast material injections when feasible and step out of the procedure room during radiographic acquisitions (digital subtraction angiography and cone-beam CT). When using angled or lateral projections, remember that the highest intensity of scattered radiation is located on the x-ray beam entrance side of the patient. When using these projections, the x-ray tube should be on the side opposite the operator whenever possible. Avoid using equipment with over-the-table x-ray tubes for interventional procedures.

*Use protective shielding*. A personal protective apron, a thyroid shield, and protective eyewear should be worn when performing fluoroscopically guided interventions. Ceiling-suspended shields can provide significant additional dose reduction, especially to unprotected areas of the head and neck. Leaded eyewear is recommended even if ceiling-suspended shields can be used continuously during the entire procedure. Under-table lead drapes reduce lower extremity dose substantially and should be used whenever possible.

*Use appropriate fluoroscopic imaging equipment*. Imaging systems optimized for one type of procedure or body part may be suboptimal for others. Using fluoroscopy equipment under suboptimal conditions frequently results in increased radiation dose. Furthermore, high radiation dose procedures should be performed with fluoroscopic systems that incorporate recommended dose-reduction technology and comply with the most current International Electrotechnical Commission (IEC) standard [133]. Encourage your institution to purchase this kind of equipment for interventional laboratories.

*Use guidance tools when available*. Biopsy planning and navigation systems, marking vascular origins with fluoroscopy overlays during stent placement, and vessel tracking to identify the course of small vessels can all be valuable guidance tools for interventional procedures.

**Radioembolization**

Details of the radioembolization pre-procedure, procedure, and post-procedure processes are provided elsewhere [134, 135]. In addition to occupational exposure from the fluoroscopic component of the procedure, interventional radiologists and other staff are exposed during transcatheter delivery of radioactive microspheres into the hepatic artery. Nurses are exposed during and after the procedure until patient discharge, though exposure from the radiopharmaceutical itself is very low after the procedure and well below any regulatory thresholds.

Procedures and safety measures are required to protect operators and other healthcare workers from occupational radiation exposure during radioembolization procedures. The facilities and procedures required depend in part on national regulations. In Europe and the U.S., specific licenses are required for handling and administrating radioactive material [134, 136]. The facility where treatment is administered must have appropriate personnel and radiation safety equipment, as well as procedures for waste handling and disposal, and the monitoring, controlling, and handling of potential contamination [135]. The instructions provided by the manufacturer should be followed. Reported equivalent doses (Hp(10)) are less than 2 μSv/GBq for both glass and resin 90Y microspheres for preparation and injection [11].

After radioembolization is completed, all exposed and possibly exposed medical products are placed into a separate decontamination container. All personnel in the fluoroscopy room are tested for contamination before leaving. The room is measured for any residual contamination before being cleared by radiation safety personnel before the next procedure [134]. In some countries this is a regulatory requirement.

Recommendations to minimize finger and hand exposure are included in the guideline [1]. Reported finger exposure for the operator injecting the microspheres is 14.0 ± 7.9 μSv/GBq with ^90^Y glass microspheres and 235.5 ± 156 μSv/GBq for ^90^Y resin microspheres [137]. With ^166^Ho, reported whole-body doses were less than 3 μSv/GBq and maximum finger dose for the operator was 2.5 ± 0.3 × 10^3^ μSv/GBq (2.5 ± 0.3 μSv/MBq) [138].

A finger (ring) dosimeter should be worn on the index finger of the hand closer to the radiation source. Ring dosimeter doses from radioembolization may be severely underestimated due to their limited sensitivity to beta emissions, the small distances between the beta source and the skin, and the high dose gradients involved [139]. To account for this, measured dose rates can be complemented by an assessment of theoretical dose rates using phantoms and dedicated software [135, 140].

Immediately after hepatic radioembolization with ^90^Y microspheres, the radiation field at 1 m from the patient’s abdomen is 1.14 μSv/h/GBq [141]. This gives 3 μSv/h for typical 2.6 GBq glass microsphere administration, and 1.8 μSv/h for 1.6 GBq resin microspheres. Another study determined mean equivalent dose rates of 1.1 mSv/h at 1 m for ^90^Y resin spheres and 2.4 mSv/h at 1 m for ^90^Y glass spheres [142]. For ^166^Ho, external exposure at 1m ranges from 8 to 60 μSv/h [143].

**CT-guided Interventions**

From an occupational protection point of view, intermittent CT is preferable to CT fluoroscopy [144]. By stepping into the control area during longer spiral scans (e.g., pre-procedural planning scan and post-procedure scan) the operator and staff take advantage of structural shielding in the walls and windows of the procedure room. This limits the time during which they are directly exposed to scatter radiation. An important note here is that an open doorway provides no additional protection – the operator and staff should take care to ensure they do not have a direct unprotected line of sight to the patient while X-rays are emitted [145].

Infection control requirements may influence the choice of radiation protection strategies, particularly if transit between the procedure room and control room is not possible. Individuals who must remain in the room during scanning should position themselves as far from the CT bore as possible while the X-ray source is energized, preferably off to the side of the gantry rather than at the head or foot of the patient. The gantry provides protection from radiation because the primary x-ray beam is attenuated by the CT detectors and the bulk of the gantry [144, 145]. Rolling shields can also be used to supplement protection. They are effective for protecting individuals who may need to remain in the room.

A lead drape or single-use sterile protective drape can be useful when performing CT fluoroscopy guided procedures [146]. A lead drape placed over the patient absorbs scattered radiation from the patient and decreases operator exposure to radiation by up to 96% [144]. However, the protection provided by such drapes depends strongly on their proximity to the area where the primary beam strikes the patient, which in CT is often very narrow. Protective drapes should be placed on the patient only after the pre-procedural planning scan has been acquired. If tube current modulation is used, the presence of a drape in the pre-procedural planning scan can drastically increase the radiation dose to both patient and operator.

When CT fluoroscopy is used to guide the procedure, the operator’s hands should never be in the CT scan plane during scanning; needle holders should always be used [144, 147]. Operators should always wear a lead apron, thyroid shield, and leaded glasses [144, 148]. A table-mounted lower body shield is useful [149]. Use of a ring dosimeter will provide the operator with information on hand exposure. Staying as far as possible from the scan plane will minimize operator dose, especially to the operator’s hands, eyes and thyroid [146]. In one study using an anthropomorphic hand voxel phantom, the dose to the operator’s hand in the CT scan plane was 18.1 mGy/sec [150]. When procedures are performed with CT fluoroscopy, long needle holders and other instruments should be used despite the potential drawbacks of these instruments, which include reduced sensitivity and tactile feedback [145]. Suspended shields are generally not useful in CT-guided interventions, and radioprotective gloves offer limited protection at the beam quality (i.e., energy) used in CT [145]. The nominal transmission through PPE is higher for CT scatter as compared to transmission from FGI procedures due to the higher operating kVp of CT radiation beams.

For most CT guided procedures, CT fluoroscopy is not necessary [144]; intermittent CT (“quick-check” or “step and shoot”) is adequate. Use of this technique substantially decreases CT times and radiation dose and avoids direct exposure of the operator’s hand [144, 147].

Adjusting CT fluoroscopy scan parameters (voltage, tube current, slice thickness) can reduce operator dose substantially [146]. Ekpo et al. [151] demonstrated a 26% increase in radiation dose to the operator’s eyes with an increase in tube voltage from 120 kV to 135 kV, and a 2-fold increase in radiation dose when the tube current was doubled from 10 mA to 20 mA. However, excessive reduction in scan parameters may result in CT images that are inadequate clinically.

If the CT scanner can reconstruct images obtained with less than a 360-degree irradiation (partial angle scanning), this should be used. When used properly, it can result in reduced operator radiation dose [152]. The lowest operator dose during partial angle CT is achieved when the operator stands on the detector side (i.e., distant from the tube) and in one study was 35% less than when 360-degree scanning was performed [152]. However, the single most effective method to reduce operator and patient dose is to minimize CT beam-on time [11, 144].

While CT fluoroscopy has a much higher dose rate than fluoroscopy with a C-arm, CT-guided procedures tend to require much less beam-on time. As a result, operator doses tend to be lower than for a typical FGI procedure. Jiang et al. estimate that, on average, an interventional radiologist would need to perform nine CT fluoroscopy procedures to receive the same occupational radiation dose received from a single C-arm fluoroscopy procedure [153].

It is sometimes necessary to perform tumor biopsy or ablation with PET-CT guidance because the lesion is not seen adequately on other modalities, or the goal is to target metabolically active portions of tumors, or there is a need to assess ablation margins during the procedure [154-156]. However, PET-CT procedures expose the operator and staff to radiation from both CT and the PET radiopharmaceutical. Options to reduce operator exposure from PET radiopharmaceuticals are limited because of the high energy (511 keV) positron-electron annihilation photons that are produced. Lead or lead-equivalent aprons are ineffective as they attenuate less than 8% of PET radiation [11, 157]. Mobile shields that provide adequate protection and do not interfere with procedures are not commercially available. Some methods that can be used include using lower or split radiopharmaceutical doses, avoiding standing next to the patient for long time, and dividing the procedure among multiple operators [156].

Fortunately, PET radiation contributes only a small fraction to patient radiation doses and personnel exposure during PET-CT-guided procedures [11, 155]. In 21 PET-CT-guided ablations, Jiang et al. found no detectable radiation due to PET behind the operator’s thyroid shield [157]. Similarly, the nurses and anesthesiologist had no detectable radiation exposure from the 21 procedures. This was likely due to their limited time near the patients. However, even in the worst-case scenario of prolonged proximity to the patients, their PET radiation dose would still be only 0.02 mSv per procedure [157]. Jiang et al. also tested using a rolling shield with 25 mm lead equivalence to protect the operator in addition to the operator’s standard lead apron. They determined that the shield provided only limited benefit because of the low exposure levels due to PET.

As of 2025, PET-CT fluoroscopy with fusion of the CT images with previously obtained PET images is not generally available but likely will be in the future [158]. PET-CT fluoroscopy allows rapid acquisition and display of CT fluoroscopy images fused to intraprocedural PET images during intermittent CT fluoroscopy. It has the potential to decrease operator time near the patient, a prime determinant of occupational dose from PET radiopharmaceuticals [11]. However, its value for guidance, radiation dose reduction, and improvement in procedure success have not yet been evaluated in detail.

**Training**

Individuals who participate in interventional radiology procedures need appropriate initial and periodic training [133, 159]. These individuals can be divided into two groups. One group includes those whose clinical responsibilities include patient radiation dose management before, during, or after procedures; the second includes all other staff in the room during a procedure. These groups are professionally diverse, including physicians, radiographers, nurses, surgical assistants, and others. Training should be based on their responsibilities in the procedure room [133, 160]. **Table 4** provides a summary. All physicians who perform interventional procedures are in group A. Other operators (in the U.S., nurse practitioners for example) may be in Group B. Training for those whose practice includes pediatric procedures needs to include additional considerations for this population. Training is recommended for non-operators (e.g., nurses, physician assistants) whose duties include assisting with patient radiation management before, during, or after procedures (Group D) [133]. Training topics for interventional radiologists and staff are described in detail in ICRP and NCRP publications [11, 133, 159]. The International Atomic Energy Agency provides a free training program that can be downloaded at [https://www.iaea.org/resources/rpop/resources/training-material#8](https://www.iaea.org/resources/rpop/resources/training-material). A great deal of other information is available on the internet. The internet is a source of both useful information and potentially bad misinformation. Readers should view web-based resources with caution.

Initial and periodic refresher training are both needed. Lifetime certification is inappropriate because of continuing changes in technology and clinical practice. The ideal initial radiation protection curriculum is individualized and based on the students’ backgrounds and duties [159, 161]. Periodic refresher training should be a subset of the individual’s initial training. An overview of the initial training content recommended for interventional radiologists (Group A) is provided in ICRP, NCRP and European Commission documents [133, 159, 162]. Subsets of this material are appropriate for refresher training of interventionalists and for the training of Groups B-E.

Non-physician instructors, typically medical physics experts, need sufficient experience with clinical procedures to be able to provide practical advice. Facility managers need to provide multiple forms of support to this process. Managers should have sufficient training so that they understand the range of patient and staff hazards associated with FGI training topics for interventional radiologists.

Modern interventional fluoroscopy systems are extremely complex and highly sophisticated. They provide many different fluoroscopic imaging and radiographic acquisition modes (e.g., low, medium and high dose fluoroscopy, high level control mode fluoroscopy, digital cineradiography, digital subtraction angiography, cone beam computed tomography) with multiple choices of pulse rate and frame rate and different options for image postprocessing. Interventional radiologists should know, for each mode, the relative differences in patient and staff radiation doses and image quality or diagnostic information. These may vary from fluoroscope to fluoroscope, even among fluoroscopes from the same manufacturer, depending on how they were set up during installation and subsequently configured. The effect of collimation, C-arm angulation, source-to-image-receptor and source-to-skin distances, and magnification on patient and staff doses is an essential part of these training programs. The medical physics expert is best able to provide this information. Fluoroscopes from different manufacturers also differ greatly in the location, operation, and function of tableside controls. Each operator of a fluoroscope needs to be familiar with the tableside controls of every fluoroscope they use [133].

All individuals who work in an interventional radiology facility need to know and be familiar with the available radiation protection tools (e.g., protective garments, ceiling-suspended screens), the level of protection they provide, and how best to use them. This includes an understanding of the purpose, value, and proper use of personal dosimeters, whether passive or active, and the C-arm ambient dosimeter, if one is used [11].

The absence of practical, hands-on training in radiation protection is a major problem identified by health professionals involved in the medical use of ionizing radiation [163]. Virtual reality has been identified as an effective radiation safety learning tool [164]. It provides a visual and active learning experience [165, 166]. One example that has worked well with students is the set of virtual labs provided on the IAEA website (<https://www.iaea.org/resources/rpop/resources/online-training-in-radiation-protection>). The German Federal Office for Radiation Protection sponsored the development of an application that simulates a virtual interventional fluoroscopy room (<https://www.bfs.de/SharedDocs/Pressemitteilungen/BfS/EN/2024/007.html>). The software is free and available at <https://app.meditrainvr.com/>. Scattered radiation and the shielding effect of patient protection products can be visualized in the software, and the entire set-up of an interventional fluoroscopy system can be explored systematically. Commercial products that simulate interventional procedures for both training and specific case preparation have been developed and are becoming popular for training. These simulators include tools to demonstrate the influence of different parameters on the radiation dose.

**Glossary**

**Absorbed dose**: the energy imparted to matter by ionizing radiation per unit mass of irradiated material at the point of interest. In the Système Internationale (SI), the unit is J kg^–1^ with the special name gray (Gy) [15].

**Accreditation:** with respect to radiation protection training, accreditation means that a training supplier has been approved by an appropriate body to provide education or training [159].

**Cohort study:** a research study that compares a particular outcome (such as lung cancer) in groups of individuals who are alike in many ways but differ by a certain characteristic, such as occupational radiation exposure. In a cohort study setting, random errors in estimates of dose tend to drive an association toward null (no effect) rather than to artificially inflate, or induce, an association.

**Detriment**: the total harm to health experienced by an exposed group and its descendants because of the group’s exposure to a radiation source. Detriment is a multi-dimensional concept. Its principal components are the stochastic quantities: probability of attributable fatal cancer, weighted probability of attributable non-fatal cancer, weighted probability of severe heritable effects, and length of life lost if the harm occurs [3].

**Dose equivalent**: a measure of the biological damage to living tissue as a result of radiation exposure. Also known as the "biological dose”, the dose equivalent is calculated as the product of absorbed dose in tissue multiplied by a quality factor and then sometimes multiplied by other necessary modifying factors at the location of interest. The dose equivalent is expressed numerically in rems or sieverts (Sv) [167].

**Dose limit**: the value of the effective dose or the equivalent dose to individuals from planned exposure situations that shall not be exceeded [3]. The intent is to prevent the occurrence of radiation-induced tissue reactions or to limit the probability of radiation-related stochastic effects.

**Equivalent dose**: the dose in a tissue or organ T given by:

where *D*_T,R_ is the mean absorbed dose from radiation R in a tissue or organ T, and *w*_R_ is the radiation weighting factor. Since *w*_R_ is dimensionless, the unit for the equivalent dose is the same as for absorbed dose, J kg^-1^, and its special name is sievert (Sv).

**Effective dose**: the tissue-weighted sum of the equivalent doses in all specified tissues and organs of the body, given by the expression:

or

where *H*T or *w*R *D*_T,R_ is the equivalent dose in a tissue or organ, T, and *w*T is the tissue
weighting factor. the unit for effective dose is the same as for absorbed dose, J kg-1, and its special name is sievert (Sv) [3]. Effective dose (E) applies only to stochastic effects.

**Effective dose equivalent**: the sum of the products of the dose equivalent to the organ or tissue () and the weighting factors () applicable to each of the body organs or tissues that are irradiated (*H*_E_ = Σ) [167].

**Equivalent dose: (*H*_T_):** mean absorbed dose in a tissue or organ (*D*_T,R_) weighted by the radiation weighting factor (*w*_R_ ) for the type and energy of radiation incident on the body:

The unit of organ equivalent dose is J kg^-1^ and has the special name sievert (Sv).

**Excess relative risk**: the rate of disease in an exposed population divided by the rate of disease in an unexposed population, minus 1.0. This is often expressed as the excess relative risk per Gy or per Sv [3]. (See also relative risk.)

**Fluoroscopically-guided interventional procedure**: an interventional diagnostic or therapeutic procedure performed via percutaneous or other access routes, usually with local anesthesia or intravenous sedation, which uses external ionizing radiation in the form of fluoroscopy to: localize or characterize a lesion, diagnostic site, or treatment site; monitor the procedure; and control and document therapy [15].

**Gray**: the special name for the SI unit of absorbed dose: 1 Gy = 1 J kg^-1^ [3].

**K-edge**:  the binding energy of the innermost electron shell (K-shell) of an atom. There is a marked increase in x-ray absorption of X-rays whose energy is just above the K-edge due to the photoelectric effect.

**Interventional Radiology**: the medical specialty that uses image guided techniques to diagnose, treat, follow up and palliate a broad range of pathologies [168].

**Maximum permissible dose**: in the U.S., a regulatory dose limit.

**Occupational radiation exposure**: radiation exposures to individuals that are incurred in the workplace because of situations that can reasonably be regarded as being the responsibility of management (radiation exposures associated with medical diagnosis of or treatment for the individual are excluded) [15].

**Odds ratio**: the ratio of the odds of an event occurring in the exposed group to the odds of the event occurring in the non-exposed group. It is commonly used in case-control studies, where the incidence rates of the outcome are not directly measured, but given the outcome the odds of exposure can be calculated.

**Operational quantities**: quantities used in practical applications for monitoring and investigating situations involving external exposure. They are defined for measurements and assessment of doses in the body [3].

**Personal Protective Equipment**: garments and devices worn to protect against radiation exposures, such as aprons, thyroid shields and leaded eyewear.

**Posture**: the spatial arrangements of body parts as they align to perform a task

**Rad**: a unit of absorbed dose. One rad is equal to an absorbed dose of 100 ergs/gram or 0.01 joule/kilogram (0.01 gray) [169]. For x-rays and gamma rays, 1 rad = 1 rem = 10 mSv.

**Relative risk**: the ratio of the probability of an event occurring in the exposed group to the probability of the event occurring in the non-exposed group. It is typically used in cohort studies and randomized controlled trials, where the incidence of an outcome can be measured directly.

**rem (Roentgen equivalent man)**: a special unit of any of the quantities expressed as dose equivalent. The dose equivalent in rems is equal to the absorbed dose in rads multiplied by the quality factor (1 rem = 0.01 sievert) [169]. The quality factor for x-rays is 1.

**Sievert**: The special name for the SI unit of equivalent dose, effective dose, and operational dose quantities. The unit is joule per kilogram (J kg^-1^).

**Stochastic effect**: malignant disease and heritable effects for which the probability of an effect occurring, but not its severity, is regarded as a function of dose without threshold [3].

**Tissue reaction**: Injury in populations of cells, characterized by a threshold dose and an increase in the severity of the reaction as the dose is increased further. Also termed tissue reaction. In some cases, tissue reactions are modifiable by post-irradiation procedures including biological response modifiers [3]. These were previously called ‘deterministic effects’.

**Tissue weighting factor**: the dimensionless factor by which equivalent dose is weighted to represent the relative contribution of that tissue or organ to the total radiation detriment resulting from uniform irradiation of the body. The *w*_T_s are judgment values grouped by organs and tissues in the interest of simplicity and rounded to sum to 1.0 [170].

**Training:** applied knowledge and practical aspects of a topic that result in improved efficiency and productivity [159]**.**

**Uncertainty**: lack of sureness or confidence in predictions of models or results of measurements [170].

**References**

1. Miller DL, Vano E, Balter S, Kitahara CM, Wunderle K, Reimer P, et al. Occupational protection in interventional radiology. A joint guideline of the Cardiovascular and Interventional Radiology Society of Europe and the Society of Interventional Radiology. Cardiovasc Intervent Radiol. 2026. <https://doi.org/10.1007/s00270-025-04195-4>

2. ICRU. Operational quantities for external radiation exposure. Report 95. Journal of the ICRU. 2020;20(1):1–130.

3. ICRP. The 2007 Recommendations of the International Commission on Radiological Protection. ICRP publication 103. Ann ICRP. 2007;37(2-4):1–332.

4. ICRP. 1990 Recommendations of the International Commission on Radiological Protection. Publication 60. Ann ICRP. 1991;21(1-3):1–201.

5. NCRP. Use of personal monitors to estimate effective dose equivalent and effective dose to workers for external exposure to low-LET radiation. NCRP Report No. 122. Bethesda, MD: National Council on Radiation Protection and Measurements; 1995.

6. NCRP. Uncertainties in the measurement and dosimetry of external radiation. NCRP Report No. 158. Bethesda, MD: National Council on Radiation Protection and Measurements; 2008.

7. Poudel S, Weir L, Dowling D, Medich DC. Changes in occupational radiation exposures after incorporation of a real-time dosimetry system in the interventional radiology suite. Health Phys. 2016;111(2 Suppl 2):S166–71.

8. Koch A, Gruber-Rouh T, Zangos S, Eichler K, Vogl T, Basten L. Radiation protection in CT-guided interventions: does real-time dose visualisation lead to a reduction in radiation dose to participating radiologists? A single-centre evaluation. Clin Radiol. 2024;79(6):e785–e90.

9. Lundvall L-L, Sandborg M. Occupational doses in interventional angiography after radiological protection training and use of a real-time direct display dosimeter. J Radiol Prot. 2022;42(3).

10. Vano E, Fernandez-Soto JM, Ten JI, Sanchez Casanueva RM. Occupational and patient doses for interventional radiology integrated into a dose management system. The British Journal of Radiology. 2023;96(1143):20220607.

11. ICRP. Occupational radiological protection in interventional procedures. ICRP Publication 139. Ann ICRP. 2018;47(2):1–118.

12. Sánchez RM, Fernández D, Vañó E, Fernández JM. Managing occupational doses with smartphones in interventional radiology. Med Phys. 2021;48(10):5830–6.

13. Sailer AM, Paulis L, Vergoossen L, Wildberger JE, Jeukens CRLPN. Optimizing staff dose in fluoroscopy-guided interventions by comparing clinical data with phantom experiments. Journal of Vascular and Interventional Radiology. 2019;30(5):701–8.

14. Vano E, Sanchez CRM, Fernandez SJM, Ten Moron JI. Alerts to improve occupational protection during interventional radiology. More attention is needed for simple but frequent procedures. Physica Medica. 2024;121:103361.

15. NCRP. Radiation dose management for fluoroscopically guided interventional medical procedures. NCRP Report No. 168. Bethesda, MD: National Council on Radiation Protection and Measurements; 2010.

16. Carinou E, Kollaard R, Stankovic Petrovic J, Ginjaume M. A European survey on the regulatory status for the estimation of the effective dose and the equivalent dose to the lens of the eye when radiation protection garments are used. J Radiol Prot. 2019;39(1):126–35.

17. Borrego D, Kitahara CM, Balter S, Yoder C. Occupational doses to medical staff performing or assisting with fluoroscopically guided interventional procedures. Radiology. 2020;294(2):353–9.

18. Vora AN, Hermiller JB, Gupta R, Goldsweig AM, Ephrem G, Al-Azizi K, et al. Variability in state-level regulations regarding occupational radiation exposure. Journal of the Society for Cardiovascular Angiography & Interventions. 2025;4(6):103597.

19. Tamura M, Kawamoto T, Ishifuro M, Tamura T, Masumoto Y, Kenjo M, et al. Radio frequency identification gate system to identify misused personal dosimeters. J Med Syst. 2023;47(1):107.

20. Fan S, Zhou W, Li M, Wang T, Deng J, Sun Q. Study on the assessment method of occupational radiation dose to interventional radiology staff wearing two personal dosimeters in China for the period 2015-2021. Health Phys. 2025.

21. WHO. Efficacy and radiation safety in interventional radiology. Geneva: World Health Organization; 2000.

22. Mussmann B, Larsen TR, Godballe M, Abdi AJ, Kantsø A, Jakobsen AR, et al. Radiation dose to multidisciplinary staff members during complex interventional procedures. Radiography. 2024;30(2):512–6.

23. Sailer AM, Paulis L, Vergoossen L, Kovac AO, Wijnhoven G, Schurink GW, et al. Real-time patient and staff radiation dose monitoring in IR practice. Cardiovasc Intervent Radiol. 2017;40(3):421–9.

24. Nessipkhan A, Matsuda N, Takamura N, Oriuchi N, Ito H, Kiguchi M, et al. Occupational radiation exposure among medical personnel in university and general hospitals in Japan. Japanese Journal of Radiology. 2024;42(9):1067–79.

25. Borrego D, Yoder C, Balter S, Kitahara CM. Collar badge lens dose equivalent values among United States physicians performing fluoroscopically guided interventional procedures. Journal of Vascular and Interventional Radiology. 2022;33(3):219–24.

26. Boice J, Jr., Dauer LT, Kase KR, Mettler FA, Jr., Vetter RJ. Evolution of radiation protection for medical workers. The British Journal of Radiology. 2020;93(1112):20200282.

27. DiSantis DJ. Early American radiology: the pioneer years. AJR Am J Roentgenol. 1986;147(4):850–3.

28. Brodsky A, Kathren RL. Historical development of radiation safety practices in radiology. Radiographics. 1989;9(6):1267–75.

29. Henshaw PS. Biologic significance of the tolerance dose in x-ray and radium protection. JNCI: Journal of the National Cancer Institute. 1941;1(6):789–805.

30. Linet MS, Kim KP, Miller DL, Kleinerman RA, Simon SL, de Gonzalez AB. Historical review of occupational exposures and cancer risks in medical radiation workers. Radiat Res. 2010;174(6):793–808.

31. Berrington de Gonzalez AB, A; Rajaraman, P; Schubauer-Berigan, M. Chapter 13: Ionizing Radiation. In: Thun L, Cerhan, Haiman, Schottenfeld, editor. Cancer Epidemiology and Prevention, 4th Edition: Oxford University Press; 2018. p. 227–48.

32. Gilbert ES, Little MP, Preston DL, Stram DO. Issues in interpreting epidemiologic studies of populations exposed to low-dose, high-energy photon radiation. Journal of the National Cancer Institute Monographs. 2020;2020(56):176–87.

33. Brenner DJ, Doll R, Goodhead DT, Hall EJ, Land CE, Little JB, et al. Cancer risks attributable to low doses of ionizing radiation: assessing what we really know. Proceedings of the National Academy of Science of the United States of America. 2003;100(24):13761–6.

34. Lee WJ, Bang YJ, Cha ES, Kim YM, Cho SB. Lifetime cancer risks from occupational radiation exposure among workers at interventional radiology departments. Int Arch Occup Environ Health. 2021;94(1):139–45.

35. Lee WJ, Jang EJ, Kim KS, Bang YJ. Underestimation of radiation doses by compliance of wearing dosimeters among fluoroscopically-guided interventional medical workers in Korea. International Journal of Environmental Research and Public Health. 2022;19(14).

36. Baudin C, Vacquier B, Thin G, Chenene L, Guersen J, Partarrieu I, et al. Radiation protection in a cohort of healthcare workers: knowledge, attitude, practices, feelings and IR-exposure in French hospitals. J Radiol Prot. 2024;44(2).

37. Sharkey AR, Gambhir P, Saraskani S, Walker R, Hajilou A, Bassett P, et al. Occupational radiation exposure in doctors: an analysis of exposure rates over 25 years. The British Journal of Radiology. 2021:20210602.

38. Lim H, Linet MS, Van DME, Miller DL, Simon SL, Sigurdson AJ, et al. Changing patterns in the performance of fluoroscopically guided interventional procedures and adherence to radiation safety practices in a U.S. cohort of radiologic technologists. AJR Am J Roentgenol. 2016;207(6):1350–9.

39. NCRP. Ionizing radiation exposure of the population of the United States. NCRP Report No. 160. Bethesda, MD: National Council on Radiation Protection and Measurements; 2009 2009.

40. NCRP. Implications of recent epidemiologic studies for the linear nonthreshold model and radiation protection. Commentary No. 27. Bethesda, MD: National Council on Radiation Protection and Measurements; 2018.

41. Shore RE, Beck HL, Boice JD, Caffrey EA, Davis S, Grogan HA, et al. Implications of recent epidemiologic studies for the linear nonthreshold model and radiation protection. J Radiol Prot. 2018;38(3):1217–33.

42. ICRP. Low-dose extrapolation of radiation-related cancer risk. Publication 99. Ann ICRP. 2006;35(4):1–140.

43. Laurier D, Billarand Y, Klokov D, Leuraud K. The scientific basis for the use of the linear no-threshold (LNT) model at low doses and dose rates in radiological protection. J Radiol Prot. 2023;43(2).

44. UNSCEAR. Sources, effects and risks of ionizing radiation. UNSCEAR 2019 Report. Scientific Annex A. Evaluation of selected health effects and inference of risk due to radiation exposure. New York: United Nations; 2019.

45. Hauptmann M, Daniels RD, Cardis E, Cullings HM, Kendall G, Laurier D, et al. Epidemiological studies of low-dose ionizing radiation and cancer: summary bias assessment and meta-analysis. Journal of the National Cancer Institute Monographs. 2020;2020(56):188–200.

46. Kitahara CM, Berrington de Gonzalez A, Bouville A, Brill AB, Doody MM, Melo DR, et al. Association of radioactive iodine treatment with cancer mortality in patients with hyperthyroidism. JAMA Intern Med. 2019;179(8):1034–42.

47. Leuraud K, Richardson DB, Cardis E, Daniels RD, Gillies M, O'Hagan JA, et al. Ionising radiation and risk of death from leukaemia and lymphoma in radiation-monitored workers (INWORKS): an international cohort study. The Lancet Haematology. 2015;2(7):e276–e81.

48. Little MP, Azizova TV, Richardson DB, Tapio S, Bernier MO, Kreuzer M, et al. Ionising radiation and cardiovascular disease: systematic review and meta-analysis. BMJ. 2023;380:e072924.

49. Richardson DB, Leuraud K, Laurier D, Gillies M, Haylock R, Kelly-Reif K, et al. Cancer mortality after low dose exposure to ionising radiation in workers in France, the United Kingdom, and the United States (INWORKS): cohort study. BMJ. 2023;382:e074520.

50. Surveillance Epidemiology and End Results (SEER) Program. DevCan database: "SEER 22 Incidence and Mortality, 2000-2021 (2020 Excluded)" http:// [www.seer.cancer.gov](file://C:\Users\morgan\AppData\Local\Microsoft\Windows\INetCache\Content.Outlook\BR0C3DGL\www.seer.cancer.gov). Bethesda, MD: National Cancer Institute, Division of Cancer Control and Population Sciences, Surveillance Research Program, Surveillance Systems Branch. Underlying mortality data provided by NCHS ([www.cdc.gov/nchs](file://C:\Users\morgan\AppData\Local\Microsoft\Windows\INetCache\Content.Outlook\BR0C3DGL\www.cdc.gov\nchs)).

51. Preston DL, Kitahara CM, Freedman DM, Sigurdson AJ, Simon SL, Little MP, et al. Breast cancer risk and protracted low-to-moderate dose occupational radiation exposure in the US Radiologic Technologists Cohort, 1983-2008. Br J Cancer. 2016;115(9):1105–12.

52. Velazquez-Kronen R, Gilbert ES, Linet MS, Moysich KB, Freudenheim JL, Wactawski-Wende J, et al. Lung cancer mortality associated with protracted low-dose occupational radiation exposures and smoking behaviors in U.S. radiologic technologists, 1983-2012. Int J Cancer. 2020;147(11):3130–8.

53. Lee T, Sigurdson AJ, Preston DL, Cahoon EK, Freedman DM, Simon SL, et al. Occupational ionising radiation and risk of basal cell carcinoma in US radiologic technologists (1983-2005). Occup Environ Med. 2015;72(12):862–9.

54. Kitahara CM, Linet MS, Balter S, Miller DL, Rajaraman P, Cahoon EK, et al. Occupational radiation exposure and deaths from malignant intracranial neoplasms of the brain and CNS in U.S. radiologic technologists, 1983-2012. AJR Am J Roentgenol. 2017:1–7.

55. Kitahara CM, Preston DL, Neta G, Little MP, Doody MM, Simon SL, et al. Occupational radiation exposure and thyroid cancer incidence in a cohort of U.S. radiologic technologists, 1983-2013. Int J Cancer. 2018;143(9):2145–9.

56. Linet MS, Little MP, Kitahara CM, Cahoon EK, Doody MM, Simon SL, et al. Occupational radiation and haematopoietic malignancy mortality in the retrospective cohort study of US radiologic technologists, 1983-2012. Occup Environ Med. 2020;77(12):822–31.

57. Lopes J, Baudin C, Rousseau F, Roy H, Lestaevel P, Caër-Lorho S, et al. Central nervous system tumours and occupational ionising radiation exposure: a nested case-control study among the ORICAMs cohort of healthcare workers in France. BMJ Open. 2024;14(6):e084285.

58. Lee WJ, Preston DL, Cha ES, Ko S, Lim H. Thyroid cancer risks among medical radiation workers in South Korea, 1996-2015. Environmental Health. 2019;18(1):19.

59. Lee WJ, Ko S, Bang YJ, Choe SA, Choi Y, Preston DL. Occupational radiation exposure and cancer incidence in a cohort of diagnostic medical radiation workers in South Korea. Occupational and Environmental Medicine. 2021;78(12):876–83.

60. Linet MS, Kitahara CM, Ntowe E, Kleinerman RA, Gilbert ES, Naito N, et al. Mortality in U.S. physicians likely to perform fluoroscopy-guided interventional procedures compared with psychiatrists, 1979 to 2008. Radiology. 2017;284(2):482–94.

61. Simpson AN, Sutradhar R, McArthur E, Tanuseputro P, Bharatha A, Ray JG. Exposure to procedural ionizing radiation and cancer risk among physicians. Occup Med. 2024;74(8):612–8.

62. Ko S, Kang S, Ha M, Kim J, Jun JK, Kong KA, et al. Health effects from occupational radiation exposure among fluoroscopy-guided interventional medical workers: a systematic review. Journal of Vascular and Interventional Radiology. 2018;29(3):353–66.

63. Roguin A, Goldstein J, Bar O, Goldstein JA. Brain and neck tumors among physicians performing Interventional procedures. The American Journal of Cardiology. 2013;111:1368–72.

64. Little MP, Azizova TV, Hamada N. Low- and moderate-dose non-cancer effects of ionizing radiation in directly exposed individuals, especially circulatory and ocular diseases: a review of the epidemiology. Int J Radiat Biol. 2021;97(6):782–803.

65. Velazquez-Kronen R, Borrego D, Gilbert ES, Miller DL, Moysich KB, Freudenheim JL, et al. Cataract risk in US radiologic technologists assisting with fluoroscopically guided interventional procedures: a retrospective cohort study. Occup Environ Med. 2019;76(5):317–25.

66. Jacob S, Boveda S, Bar O, Brézin A, Maccia C, Laurier D, et al. Interventional cardiologists and risk of radiation-induced cataract: Results of a French multicenter observational study. Int J Cardiol. 2013;167(5):1843–7.

67. Auvinen A, Kivela T, Heinavaara S, Mrena S. Eye lens opacities among physicians occupationally exposed to ionizing radiation. The Annals of Occupational Hygiene. 2015;59(7):945–8.

68. Scheidemann-Wesp U, Gianicolo EAL, Camara RJ, Wegener A, Buchner SE, Schwenn O, et al. Ionising radiation and lens opacities in interventional physicians: results of a German pilot study. Journal of Radiolological Protection. 2019;39(4):1041–59.

69. Vano E, Kleiman NJ, Duran A, Rehani MM, Echeverri D, Cabrera M. Radiation cataract risk in interventional cardiology personnel. Radiat Res. 2010;174(4):490–5.

70. Domienik-Andrzejewska J, Kaluzny P, Piernik G, Jurewicz J. Occupational exposure to ionizing radiation and lens opacity in interventional cardiologists. Int J Occup Med Environ Health. 2019;32(5):663–75.

71. Karatasakis A, Brilakis HS, Danek BA, Karacsonyi J, Martinez-Parachini JR, Nguyen-Trong PJ, et al. Radiation-associated lens changes in the cardiac catheterization laboratory: Results from the IC-CATARACT (CATaracts Attributed to RAdiation in the CaTh lab) study. Catheter Cardiovasc Interv. 2018;91(4):647–54.

72. Ciraj-Bjelac O, Rehani M, Minamoto A, Sim KH, Liew HB, Vano E. Radiation-induced eye lens changes and risk for cataract in interventional cardiology. Cardiology. 2012;123(3):168–71.

73. Andreassi MG, Piccaluga E, Guagliumi G, Del GM, Gaita F, Picano E. Occupational health risks in cardiac catheterization laboratory workers. Circ Cardiovasc Interv. 2016;9(4):e003273.

74. Little MP, Kitahara CM, Cahoon EK, Bernier MO, Velazquez-Kronen R, Doody MM, et al. Occupational radiation exposure and risk of cataract incidence in a cohort of US radiologic technologists. Eur J Epidemiol. 2018;33(12):1179–91.

75. Lopes R, Teles P, Santos J. A systematic review on the occupational health impacts of ionising radiation exposure among healthcare professionals. J Radiol Prot. 2025;45(2).

76. Little MP, Kitahara CM, Cahoon EK, Bernier MO, Velazquez-Kronen R, Doody MM, et al. Occupational radiation exposure and glaucoma and macular degeneration in the US radiologic technologists. Scientific Reports. 2018;8(1):10481.

77. Lopes J, Leuraud K, Klokov D, Durand C, Bernier MO, Baudin C. Risk of developing non-cancerous central nervous system diseases due to ionizing radiation exposure during adulthood: systematic review and meta-analyses. Brain Sciences. 2022;12(8):984.

78. Gillies M, Richardson DB, Cardis E, Daniels RD, O'Hagan JA, Haylock R, et al. Mortality from circulatory diseases and other non-cancer outcomes among nuclear workers in France, the United Kingdom and the United States (INWORKS). Radiat Res. 2017;188(3):276–90.

79. Bang YJ, Kim YM, Lee WJ. Circulatory disease mortality among male medical radiation workers in South Korea, 1996-2019. Scand J Work Environ Health. 2023;49(2):99–107.

80. Dauer LT, Walsh L, Mumma MT, Cohen SS, Golden AP, Howard SC, et al. Moon, mars and minds: evaluating Parkinson's disease mortality among U.S. radiation workers and veterans in the million person study of low-dose effects. Zeitschrift für Medizinische Physik. 2024;34(1):100–10.

81. Azizova TV, Bannikova MV, Grigoryeva ES, Rybkina VL, Hamada N. Occupational exposure to chronic ionizing radiation increases risk of parkinson's disease incidence in Russian Mayak workers. Int J Epidemiol. 2020;49(2):435–47.

82. Laurent O, Samson E, Caer-Lorho S, Fournier L, Laurier D, Leuraud K. Updated mortality analysis of SELTINE, the French cohort of nuclear workers, 1968-2014. Cancers (Basel). 2022;15(1).

83. Liu JJ, Freedman DM, Little MP, Doody MM, Alexander BH, Kitahara CM, et al. Work history and mortality risks in 90,268 US radiological technologists. Occup Environ Med. 2014;71(12):819–35.

84. Rajaraman P, Doody MM, Yu CL, Preston DL, Miller JS, Sigurdson AJ, et al. Incidence and mortality risks for circulatory diseases in US radiologic technologists who worked with fluoroscopically guided interventional procedures, 1994-2008. Occup Environ Med. 2016;73(1):21–7.

85. Morrison JJ, Jiao A, Robinson S, Jahangiri Y, Kaufman JA. Prevalence of musculoskeletal symptoms in interventional radiologists. Journal of Vascular and Interventional Radiology. 2020;31(8):1308–14.

86. Knuttinen MG, Zurcher KS, Wallace A, Doe C, Naidu SG, Money SR, et al. Ergonomics in IR. Journal of Vascular and Interventional Radiology. 2021;32(2):235–41.

87. Schlussel AT, Maykel JA. Ergonomics and musculoskeletal health of the surgeon. Clinics in Colon and Rectal Surgery. 2019;32(6):424–34.

88. Abudayyeh I, Dupont AG, Hermiller JB, Mascarenhas J, Velagapudi P, Ijioma NN, et al. Occupational health hazards in the cardiac catheterization laboratory: results of the 2023 SCAI survey. Journal of the Society for Cardiovascular Angiography & Interventions. 2025;4(4):102493.

89. Cornelis FH, Razakamanantsoa L, Ben AM, Lehrer R, Haffaf I, El-Mouhadi S, et al. Ergonomics in interventional radiology: awareness is mandatory. Medicina (Kaunas). 2021;57(5):500.

90. McQuivey KS, Deckey DG, Christopher ZK, Rosenow CS, Mi L, Spangehl MJ, et al. Surgical ergonomics and musculoskeletal pain in orthopaedic surgery residents: a multicenter survey study. Journal of the American Academy of Orthopaedic Surgeons Global Research & Reviews. 2021;5(3):e20.00119.

91. Orme NM, Rihal CS, Gulati R, Holmes DR, Jr., Lennon RJ, Lewis BR, et al. Occupational health hazards of working in the interventional laboratory: a multisite case control study of physicians and allied staff. J Am Coll Cardiol. 2015;65(8):820–6.

92. Li Y, Xu H, Li R, Cheng M, Wen C. Prevalence and risk factors of work-related musculoskeletal disorders among healthcare workers in medical radiation environments. Work. 2024;79(4):1867–77.

93. ACS. Surgical ergonomics recommendations. Chicago: American College of Surgeons; 2021. Available from: <https://www.facs.org/media/cezf1xvo/surgicalergonomicsrecommendations.pdf>.

94. Dixon RG, Khiatani V, Statler JD, Walser EM, Midia M, Miller DL, et al. Society of Interventional Radiology: occupational back and neck pain and the interventional radiologist. Journal of Vascular and Interventional Radiology. 2017;28(2):195–9.

95. Klein LW, Miller DL, Balter S, Laskey W, Haines D, Norbash A, et al. Occupational health hazards in the interventional laboratory: time for a safer environment. J Vasc Interv Radiol. 2009;20(2):147–52.

96. Miller DL, Klein LW, Balter S, Norbash A, Haines D, Fairobent L, et al. Occupational health hazards in the interventional laboratory: progress report of the Multispecialty Occupational Health Group. Journal of Vascular and Interventional Radiology. 2010;21(9):1338–41.

97. AAPM. Interoperability assessment for the commissioning of medical imaging acquisition systems. AAPM Report No. 248. Alexandria, VA: American Association of Physicists in Medicine; 2019.

98. van Veelen MA, Kazemier G, Koopman J, Goossens RH, Meijer DW. Assessment of the ergonomically optimal operating surface height for laparoscopic surgery. J Laparoendosc Adv Surg Tech A. 2002;12(1):47–52.

99. Choi HS, In H. The effects of operating height and the passage of time on the end-point performance of fine manipulative tasks that require high accuracy. Frontiers in Physiology. 2022;13:944866.

100. Shea FJ, Ziskin MC. Visual system transfer function and optimal viewing distance for radiologists. Invest Radiol. 1972;7(3):147–51.

101. Hallbeck MS, Lowndes BR, Bingener J, Abdelrahman AM, Yu D, Bartley A, et al. The impact of intraoperative microbreaks with exercises on surgeons: A multi-center cohort study. Appl Ergon. 2017;60:334–41.

102. Park AE, Zahiri HR, Hallbeck MS, Augenstein V, Sutton E, Yu D, et al. Intraoperative "micro breaks" with targeted stretching enhance surgeon physical function and mental focus: a multicenter cohort study. Ann Surg. 2017;265(2):340–6.

103. Ciekalski M, Rosół I, Filipek M, Gruca M, Hankus M, Hanslik K, et al. Work-related musculoskeletal disorders in Polish sonographers-a questionnaire study. Curr Probl Diagn Radiol. 2024;53(5):576–82.

104. Chang WD, Lin HY, Lai PT. Core strength training for patients with chronic low back pain. Journal of Physical Therapy Science. 2015;27(3):619–22.

105. Valenza MC, Rodriguez-Torres J, Cabrera-Martos I, Diaz-Pelegrina A, Aguilar-Ferrandiz ME, Castellote-Caballero Y. Results of a Pilates exercise program in patients with chronic non-specific low back pain: a randomized controlled trial. Clin Rehabil. 2017;31(6):753–60.

106. Kumar S, Prasad S, Balakrishnan B, Muthukumaraswamy K, Ganesan M. Effects of Isha Hatha yoga on core stability and standing balance. Adv Mind Body Med. 2016;30(2):4–10.

107. Domienik-Andrzejewska J, Mirowski M, Jastrzebski M, Gornik T, Masiarek K, Warchol I, et al. Occupational exposure to physicians working with a Zero-Gravity protection system in haemodynamic and electrophysiology labs and the assessment of its performance against a standard ceiling suspended shield. Radiat Environ Biophys. 2022;61(2):293–300.

108. Hout JD, Ryu J. The association between musculoskeletal disorders and lead apron use in healthcare workers: A systematic review and meta-analysis. Safety Science. 2025;181:106669.

109. Koenig AM, Froehlich L, Viniol S, Thomas RP, Mahnken AH. Occupational orthopedic problems and its relation to personal radiation protection in interventional radiology. Eur J Radiol. 2024;175:111401.

110. Koenig AM, Schweer A, Sasse D, Etzel R, Apitzsch J, Viniol S, et al. Physical strain while wearing personal radiation protection systems in interventional radiology. PLoS One. 2022;17(7):e0271664.

111. Lichliter A, Weir V, Heithaus RE, Gipson S, Syed A, West J, et al. Clinical evaluation of protective garments with respect to garment characteristics and manufacturer label information. Journal of Vascular and Interventional Radiology. 2017;28(1):148–55.

112. Ramanan B, Pizano A, Solano A, Gonugunta AS, Timaran CH, Siah M, et al. The addition of a leaded arm sleeve to leaded aprons further decreases operator upper outer quadrant chest wall radiation dose during fluoroscopically guided interventions. J Vasc Surg. 2024;79(4):948–53.

113. Choi TW, Chung JW, Kwon Y. Modified design of x-ray protective clothing to enhance radiation protection for interventional radiologists. Med Phys. 2023;50(6):3825–32.

114. Modarai B, Haulon S, Ainsbury E, Bockler D, Vano-Carruana E, Dawson J, et al. European Society for Vascular Surgery (ESVS) 2023 Clinical Practice Guidelines on Radiation Safety. Eur J Vasc Endovasc Surg. 2023;65(2):171–222.

115. Westcott LZ, Ogola GO, Rees CR. Protecting our own: a method for reducing breast radiation exposure in healthcare workers. Health Phys. 2024.

116. Lopes R, Teles P, Santos J. A systematic review of the effectiveness of leaded glasses for ensuring safety among healthcare professionals in fluoroscopy. Journal of Medical Imaging and Radiation Sciences. 2025;56(2):101848.

117. Imai S, Yamahata A, Gotanda T, Akahane M, Kawaji Y, Akagawa T, et al. Evaluation of factors influencing eye lens radiation dose while using radiation protection glasses in interventional radiology: A phantom study. Eur J Radiol. 2025;184:111943.

118. Schueler BA, Fetterly KA. Eye protection in interventional procedures. The British Journal of Radiology. 2021;94(1126):20210436.

119. Vanhavere F, Carinou E, Gualdrini G, Clairand I, Sans Merce M, Ginjaume M, et al. ORAMED: Optimization of Radiation Protection of Medical Staff. EURADOS Report 2012-02. Braunschweig, Germany: European Radiation Dosimetry Group e. V.; 2012 April 2012.

120. Katsarou M, Zwiebel B, Chowdhury RP, Shames M, Berger T, Przybyla B, et al. Experimental analysis of radiation protection offered by a novel exoskeleton-based radiation protection system versus conventional lead aprons. Journal of Vascular and Interventional Radiology. 2023;34(8):1345–52.

121. Apostolou A, Leichert HJ, Konig AM, Owczarek AD, Mahnken AH. Efficiency in radiation protection of a novel exoskeleton-based interventional radiology apron and correlation with conventional aprons. Eur J Radiol. 2025;184:111946.

122. Katsarou M, Zwiebel B, Vogler J, Shames ML, Thayer A, Chowdhurry RP, et al. StemRad MD, an exoskeleton-based radiation protection system, reduces ergonomic posture risk based on a prospective observational study. J Endovasc Ther. 2024;31(4):668–74.

123. Budošová D, Horváthová M, Bárdyová Z, Balázs T. Current trends of radiation protection equipment in interventional radiology. Radiat Prot Dosimetry. 2022;198(9-11):554–9.

124. Crowhurst JA, Tse J, Mirjalili N, Savage ML, Raffel OC, Gaikwad N, et al. Trial of a novel radiation shielding device to protect staff in the cardiac catheter laboratory. The American Journal of Cardiology. 2023;203:429–35.

125. Rizik DG, Riley RD, Burke RF, Klassen SR, Nigoghosian AM, Gosselin KP, et al. Comprehensive radiation shield minimizes operator radiation exposure and obviates need for lead aprons. Journal of the Society for Cardiovascular Angiography & Interventions. 2023;2(3):100603.

126. Smith L, Caffrey E, Wilson C. A novel shielding device for cardiac cath labs. Health Phys. 2025;128(1):52–9.

127. Riley RF, Kamen J, Tao A, Gomez-Cardona D. Comparative effectiveness of the EggNest complete shielding system to standard shielding in the cath lab. American Heart Journal Plus: Cardiology Research and Practice. 2025;54:100543.

128. Irani Z, Alexander B, Zhang D, Liu B, Ghoshhajra B, Oklu R. Novel lead-free drape applied to the x-ray detector protects against scatter radiation in the angiography suite. Journal of Vascular and Interventional Radiology. 2014;25(8):1200–8.

129. Miller DL, Vañó E, Bartal G, Balter S, Dixon R, Padovani R, et al. Occupational radiation protection in interventional radiology: a joint guideline of the Cardiovascular and Interventional Radiology Society of Europe and the Society of Interventional Radiology. Cardiovasc Intervent Radiol. 2010;33(2):230–9.

130. Fritz S, Jones AK. Guidelines for anti-scatter grid use in pediatric digital radiography. Pediatr Radiol. 2014;44(3):313–21.

131. McFadden SL, Hughes CM, Mooney RB, Winder RJ. An analysis of radiation dose reduction in paediatric interventional cardiology by altering frame rate and use of the anti-scatter grid. J Radiol Prot. 2013;33(2):433–43.

132. Ubeda C, Vano E, Gonzalez L, Miranda P. Influence of the antiscatter grid on dose and image quality in pediatric interventional cardiology x-ray systems. Catheter Cardiovasc Interv. 2013;82(1):51–7.

133. NCRP. Recommendations for stratification of equipment use and radiation safety training for fluoroscopy. Commentary No. 33. Bethesda, MD: National Council on Radiation Protection and Measurements; 2023.

134. Busse NC, Al-Ghazi MSAL, Abi-Jaoudeh N, Alvarez D, Ayan AS, Chen E, et al. AAPM Medical Physics Practice Guideline 14.a: Yttrium-90 microsphere radioembolization. Journal of Applied Clinical Medical Physics. 2023:e14157.

135. Weber M, Lam M, Chiesa C, Konijnenberg M, Cremonesi M, Flamen P, et al. EANM procedure guideline for the treatment of liver cancer and liver metastases with intra-arterial radioactive compounds. European Journal of Nuclear Medicine and Molecular Imaging. 2022;49(5):1682–99.

136. European Commission. Council Directive 2013/59/Euratom of 5 December 2013 laying down basic safety standards for protection against the dangers arising from exposure to ionising radiation, and repealing Directives 89/618/Euratom, 90/641/Euratom, 96/29/Euratom, 97/43/Euratom and 2003/122/Euratom.2014. 1–73 (17 Jan 2014) p.

137. Laffont S, Rolland Y, Ardisson V, Edeline J, Pracht M, Le SS, et al. Occupational radiation exposure of medical staff performing ^90^Y-loaded microsphere radioembolization. European Journal of Nuclear Medicine and Molecular Imaging. 2016;43(5):824–31.

138. Taleb J, Janier M, Bonazza P, Roux P, Miladi I, Goutain-Majorel C, et al. Radiation dose measurements for staff members involved in holmium-166 preclinical trial. Radiation Measurements. 2013;58:75–8.

139. Rimpler A, Barth I. Beta radiation exposure of medical staff and implications for extremity dose monitoring. Radiat Prot Dosimetry. 2007;125(1-4):335–9.

140. Becker F, Blunck C. Investigation of radiation exposure of medical staff: Measurements supported by simulations with an articulated hand phantom. Radiation Measurements. 2011;46(11):1299–302.

141. ARPANSA. Discharge of patients undergoing treatment with radioactive substances. Radiation Protection Series Publication No. 4. Yallambie, Australia: Australian Radiation Protection and Nuclear Safety Agency; 2002.

142. McCann JW, Larkin AM, Martino LJ, Eschelman DJ, Gonsalves CF, Brown DB. Radiation emission from patients treated with selective hepatic radioembolization using yttrium-90 microspheres: are contact restrictions necessary? Journal of Vascular and Interventional Radiology. 2012;23(5):661–7.

143. Prince JF, Smits ML, Krijger GC, Zonnenberg BA, van dBMA, Nijsen JF, et al. Radiation emission from patients treated with holmium-166 radioembolization. Journal of Vascular and Interventional Radiology. 2014;25(12):1956–63.

144. Sarti M, Brehmer WP, Gay SB. Low-dose techniques in CT-guided interventions. Radiographics. 2012;32(4):1109–19; discussion 19–20.

145. Jones AK, Dixon RG, Collins JD, Walser EM, Nikolic B. Best practice guidelines for CT-guided interventional procedures. Journal of Vascular and Interventional Radiology. 2018;29(4):518–9.

146. Nakatani M, Kariya S, Ono Y, Maruyama T, Ueno Y, Komemushi A, et al. Radiation exposure and protection in computed tomography fluoroscopy. Interventional Radiology. 2022;7(2):49–53.

147. Sarmento S, Pereira JS, Sousa MJ, Cunha LT, Dias AG, Pereira MF, et al. The use of needle holders in CTF guided biopsies as a dose reduction tool. Journal of Applied Clinical Medical Physics. 2018;19(1):250–8.

148. Inaba Y, Hitachi S, Watanuki M, Chida K. Occupational radiation dose to eye lenses in CT-guided interventions using MDCT-fluoroscopy. Diagnostics (Basel). 2021;11(4):646.

149. Mahnken AH, Sedlmair M, Ritter C, Banckwitz R, Flohr T. Efficacy of lower-body shielding in computed tomography fluoroscopy-guided interventions. Cardiovasc Intervent Radiol. 2012;35(6):1475–9.

150. Figueira C, Becker F, Blunck C, DiMaria S, Baptista M, Esteves B, et al. Medical staff extremity dosimetry in CT fluoroscopy: an anthropomorphic hand voxel phantom study. Phys Med Biol. 2013;58(16):5433–48.

151. Ekpo EU, Bakhshi S, Ryan E, Hogg P, McEntee MF. Operator eye doses during computed tomography fluoroscopic lung biopsy. J Radiol Prot. 2016;36(2):290–8.

152. Knott EA, Rose SD, Wagner MG, Lee FT, Jr., Radtke J, Anderson DR, et al. CT fluoroscopy for image-guided procedures: physician radiation dose during full-rotation and partial-angle CT scanning. Journal of Vascular and Interventional Radiology. 2021;32(3):439–46.

153. Knott EA, Troville JL, Reynoso CA, Rose SD, Wagner MG, Lee Jr FT, et al. Physician scatter dose in interventional CT fluoroscopy. Journal of Applied Clinical Medical Physics. 2024;25(6):e14355.

154. Shyn PB, Cubre AJ, Catalano PJ, Lee LK, Hyun H, Tuncali K, et al. F-18 FDG perfusion PET: intraprocedural assessment of the liver tumor ablation margin. Abdom Radiol (NY). 2021;46(7):3437–47.

155. Bogoni M, Cerci JJ, Cornelis FH, Nanni C, Tabacchi E, SchOder H, et al. Practice and prospects for PET/CT guided interventions. The Quarterly Journal of Nuclear Medicine and Molecular Imaging. 2021;65(1):20–31.

156. Shyn PB, Bhagavatula SK. PET/CT-guided tumor ablation, from the AJR "How We Do It" special series. AJR Am J Roentgenol. 2025;224(1):e2430952.

157. Jiang L, Jowkar N, Bhagavatula SK, Levesque VM, Walsh MF, Kapur T, et al. Positron emission tomography and computed tomography contributions to patient dose and personnel exposure to radiation during PET/CT-guided tumor ablations. Journal of Vascular and Interventional Radiology. 2022;33(10):1234–9.

158. Jiang L, Jowkar N, King F, Plishker W, Bhagavatula SK, Levesque VM, et al. PET/CT fluoroscopy during PET/CT-guided interventions: initial experience. Journal of Vascular and Interventional Radiology. 2023;34(8):1319–23.

159. ICRP. Education and training in radiological protection for diagnostic and interventional procedures. ICRP Publication 113. Ann ICRP. 2009;39(5):1–68.

160. Miller DL, Balter S. NCRP commentary no. 33-recommendations for stratification of equipment use and radiation safety training for fluoroscopy. J Radiol Prot. 2024;44(2):023001.

161. NCRP. Radiation protection for procedures performed outside the Radiology Department. NCRP Report No. 133. Bethesda, MD: National Council on Radiation Protection and Measurements; 2000.

162. European Commission. Radiation Protection N° 175. Guidelines on radiation protection education and training of medical professionals in the European Union. Luxembourg: Publications Office of the European Union; 2014.

163. Santos J, Foley S, Andersson J, Figueiredo JP, Hoeschen C, Damilakis J, et al. Education and training in radiation protection in Europe: results from the EURAMED Rocc-n-Roll project survey. Insights Imaging. 2023;14(1):55.

164. Mwangi W, Tanaka Y. Comparative effectiveness of immersive virtual reality and traditional didactic training on radiation safety in medical professionals: a crossover study. Journal of Medical Radiation Sciences. 2025.

165. Rainford L, Tcacenco A, Potocnik J, Brophy C, Lunney A, Kearney D, et al. Student perceptions of the use of three-dimensional (3-D) virtual reality (VR) simulation in the delivery of radiation protection training for radiography and medical students. Radiography. 2023;29(4):777–85.

166. Noguchi K, Fujibuchi T, Han D. Effectiveness of radiation protection educational material during angiography using visualization of scattered radiation by augmented reality technique. Radiol Phys Technol. 2025.

167. U.S. Nuclear Regulatory Commission. 10 CFR 20.1003 Definitions. Washington, DC: Nuclear Regulatory Commission.

168. IAEA. Establishing and improving interventional radiology. Vienna: International Atomic Energy Agency; 2023 October 2023.

169. U.S. Nuclear Regulatory Commission. 10 CFR 20.1004 Units of radiation dose. Washington, DC: Nuclear Regulatory Commission.

170. NCRP. Management of exposure to ionizing radiation: radiation protection guidance for the United States. Report No. 180. Bethesda, Maryland: National Council on Radiation Protection and Measurements; 2018.

**Table 1. Cancer incidence and mortality in the U.S. population, by year, calculated using data from the Surveillance Epidemiology and End Results (SEER) Program [50]**

| **Years** | **Probability of developing cancer (%)** |
| --- | --- |
| 2018-2021 (2020 excluded) ^a^ | 39.33 |
| 2015-2017 | 40.85 |
| 2012-2014 | 40.91 |
| 2009-2011 | 42.81 |
|  |  |
|  | **Probability of dying of cancer (%)** |
| 2018-2021 (2020 excluded) | 17.89 |
| 2015-2017 | 19.53 |
| 2012-2014 | 20.36 |
| 2009-2011 | 20.87 |

^a^ 2020 data excluded because of the effect of COVID-19

**Table 2. Results from selected large worker cohorts that examined cancer risks associated with occupational radiation exposure. Statistically significant results are shown in bold.**

| First author, publication year | Cohort | Outcome (N) | Measure of effect |
| --- | --- | --- | --- |
| Richardson, 2023 [49] | INWORKS (multinational cohort of >300,000 monitored nuclear industry workers) | Deaths due to solid cancer (28,089)  Deaths due to lung cancer (19,950) | **ERR/100 mGy: 0.05 (90% CI: 0.03, 0.08)**  **ERR/100 mGy: 0.04 (90% CI: 0.02, 0.07)** |
| Leuraud, 2015 [47] | INWORKS (multinational cohort of >300,000 monitored nuclear industry workers) | Deaths due to leukemia, not including chronic lymphocytic leukemia (1,791) | **ERR/100 mGy: 0.30 (90% CI: 0.12, 0.52)** |
| Preston, 2016 [51] | USRT (cohort of >110,000 U.S. radiographers) | Incident breast cancer (1,922)  Deaths due to breast cancer (586) | **ERR/100 mGy: 0.07 (95% CI: 0.005, 0.19)**  **ERR/100 mGy: 0.31 (95% CI: 0.11, 0.67)** |
| Velazquez-Kronen, 2020 [52] | USRT (cohort of >110,000 U.S. radiographers) | Deaths due to lung cancer (1,090) | ERR/100 mGy, overall:−0.02 (95% CI: <0-0.13)  By smoking intensity:  **<20 pack-years: 0.41 (95% CI: 0.01, 1.15**)  ≥20 pack-years: −0.03 (95% CI: <0-0.15) |
| Linet, 2020 [56] | USRT (cohort of >110,000 U.S. radiographers) | Deaths due to leukemia, not including chronic lymphocytic leukemia (155) | ERR/100 mGy: 0.05 (95% CI <-0.09, 0.24) |
| Kitahara, 2017 [54] | USRT (cohort of >110,000 U.S. radiographers) | Brain cancer mortality (193) | ERR/100 mGy: 0.1 (95% CI < –0.3, 1.5) |
| Kitahara, 2018 [55] | USRT (cohort of >110,000 U.S. radiographers) | Incident thyroid cancer (476) | ERR/100 mGy: -0.05 (95% CI <–0.10, 0.34) |
| Lee, 2015 [53] | USRT (cohort of >110,000 U.S. radiographers) | Incident basal cell carcinoma (3,615) | ERR/100 mGy: -0.001 (95% CI: -0.004, 0.005) |
| Linet, 2017 [60] | 45634 “exposed” U.S. physicians (>90% males) who performed interventional radiology procedures (37311 cardiologists, 5520 interventional radiologists, and 2803 neuroradiologists) and 64,401 “unexposed” U.S. psychiatrists | Deaths due to cancer (1140 in exposed males; 2154 in unexposed males) | Exposed vs. unexposed, RR (males): 0.92 (95% CI: 0.85, 0.99) ^a^ |
| Simpson, 2024 [61] | 1265 cancer cases and 5772 controls. |  | OR per 1000 ionizing radiation procedures: 1.02 (95% CI: 0.99, 1.05) |

*ERR* excess relative risk; *RR* relative risk; *OR* Odds ratio; *CI* Confidence interval; *USRT* U.S. Radiologic Technologists Study

ERR per unit dose is approximately equal to the RR per unit dose minus 1.

^a^ No significantly increased risks observed for specific causes of cancer death

**Table 3. Results from selected large worker cohorts examining tissue reactions associated with occupational radiation exposure. Statistically significant results are shown in bold.**

| **First author, publication year** | **Cohort** | **Outcome (N)** | **Measure of effect** |
| --- | --- | --- | --- |
| Little, 2018 [74] | USRT (cohort of >110,000 U.S. radiographers) | Incident cataract (12,336)  Surgically-removed cataracts (5,509) | **ERR/100 mGy: 0.069 (95% CI: 0.027, 0.116; lagged 5 years)**  **ERR/100 mGy: 0.034 (95% CI -0.019, 0.097; lagged 5 years)** |
| Velazquez-Kronen, 2019 [65] | USRT (cohort of >110,000 U.S. radiographers) | Incident cataract (9,372) | **Ever vs never worked with interventional radiology procedures, RR: 1.18 (95% CI 1.11 to 1.25)**  **Higher risk with greater procedure frequency, particularly when standing <3 feet of the patient** |
| Little, 2018 [76] | USRT (cohort of >110,000 U.S. radiographers) | Incident glaucoma (1,631)  Incident macular degeneration (1,331) | ERR/100 mGy: -0.06 (95% CI: -0.15, 0.06)  ERR/100 mGy: 0.03 (95% CI: -0.03, 0.13) |
| Gillies, 2017 [78] | INWORKS (multinational cohort of >300,000 monitored nuclear industry workers) | Deaths due to circulatory disease (25,570)  Deaths due to respiratory disease (5,291)  Deaths due to digestive disease (2,180)  Deaths due to external causes (4,451) | **ERR/100 mSv: 0.02 (90% CI: 0.01, 0.04) ^a^**  ERR/100 mSv: 0.01 (90% CI: -0.02, 0.05)  ERR/100 mSv: 0.01 (90% CI: -0.04, 0.07)  ERR/100 mSv: -0.01 (90% CI: -0.06, 0.05) |
| Bang et al., 2023 [79] | Cohort of >50,000 Korean male diagnostic medical radiation workers | Deaths due to circulatory disease (320) | ERR/100 mGy: 0.85 (95% CI: -0.11, 1.82) ^b^ |
| Linet, 2017 [60] | 45634 “exposed” U.S. physicians (>90% male) who performed interventional radiology procedures (including 37311 cardiologists, 5520 interventional radiologists, and 2803 neuroradiologists)  and 64,401 “unexposed” U.S. psychiatrists | Deaths due to cardiovascular diseases (1299 in exposed males; 2642 in unexposed males)  Deaths due to neurological and mental conditions (149 in exposed males/280 in unexposed males) | Exposed vs unexposed, RR (males): 0.87 (95% CI: 0.82, 0.93) ^c^  Exposed vs unexposed, RR (males): 0.94 (95% CI: 0.77, 1.15) |
| Dauer, 2024 [80] | 517,608 workers from 6 cohorts within the Million Person Study | Deaths due to Parkinson’s disease | ERR/100 mGy: 0.17 (95% CI: 0.05, 0.29) |
| Azizova, 2020 [81] | 22,377 Russian nuclear production facility workers | Incident Parkinson’s disease (300) | ERR/100 mGy: 0.10 (95% CI: 0.06, 0.16) |
| Laurent, 2022 [82] | 80,348 French nuclear workers | Deaths due to Parkinson’s disease (124)  Deaths due to dementia/Alzheimer’s disease (269) | ERR/100 mGy: -0.13 (95% CI: n.e., 0.74)  ERR/100 mGy: 0.96 (95% CI: 0.31, 1.87) |

*ERR* Excess relative risk*, RR* Relative risk*, n.e.* Not estimable*, CI* Confidence interval

ERR per unit dose is approximately equal to the RR per unit dose minus 1.

^a^ Similar results for ischemic heart disease (ERR/100 mSv: 0.02 (90% CI: 0.00, 0.04)) and cerebrovascular disease (ERR/100 mSv: 0.05 (90% CI: 0.01, 0.09))

^b^ Similar results for ischemic heart disease (ERR/100 mGy: 1.18, 95% CI -0.69, 3.05) and cerebrovascular disease (ERR/100 mGy: 0.23, 95% CI -0.48, 0.94)

^c^ No increased risks for specific types of cardiovascular disease deaths

**Table 4. Training groups (modified with permission from [133])**

| **Group** | **Individuals in the Group** | **Staff Risk for** | **Patient Risk  per procedure of** | |
| --- | --- | --- | --- | --- |
|  |  | **Stochastic Injury** | **Stochastic Injury** | **Tissue Reaction** |
| A | Operators^a^ performing potentially high patient dose procedures | Minimal  to minor | Low | Possible |
| B | Operators^a^ performing intermediate patient dose procedures | Minimal  to minor | Minimal  to minor | Highly Unlikely |
| C | Operators^a^ performing only low patient dose procedures | Minimal | Negligible | Not Expected |
| D | Other individuals^a^ assisting with patient radiation management^b^ | Minimal | N/A | N/A |
| E | All other individuals in the fluoroscopic room while procedures are in progress^b^ | Minimal | N/A | N/A |
| F | All other individuals in the facility who might encounter a fluoroscope or enter a fluoroscopic room | Simple instruction, integrated into the facility’s general safety training | | |
| ^a^ This also includes those who supervise these individuals  ^b^ Content should be adjusted to meet the individual’s professional background and the needs of the highest risk procedures that they support | | | | |
